# Bcl6 is a subset defining transcription factor of Lymphoid Tissue inducer-like ILC3

**DOI:** 10.1016/j.celrep.2023.113425

**Published:** 2023-11-10

**Authors:** Roser Tachó-Piñot, Christopher T. Stamper, James I. King, Veronika Matei-Rascu, Erin Richardson, Zhi Li, Luke B. Roberts, John W. Bassett, Felipe Melo-Gonzalez, Rémi Fiancette, I-Hsuan Lin, Alexander Dent, Yohsuke Harada, Conor Finlay, Jenny Mjösberg, David R. Withers, Matthew R. Hepworth

**Affiliations:** 1 Lydia Becker Institute of Immunology and Inflammation, University of Manchester, M13 9PL, United Kingdom; 2 Division of Immunology, Immunity to Infection and Respiratory Medicine, School of Biological Sciences, Faculty of Biology, Medicine and Health, Manchester Academic Health Science Centre, University of Manchester, M13 9PL, United Kingdom; 3 Center for Infectious Medicine, Department of Medicine Huddinge, Karolinska Institutet, Stockholm, Sweden; 4 Medical unit for lung and allergy diseases, Karolinska University Hospital, Stockholm, Sweden; 5 Institute of Immunology and Immunotherapy, College of Medical and Dental Sciences, University of Birmingham, Birmingham, B15 2TT, United Kingdom; 6 Bioinformatics Core Facility, University of Manchester, Manchester, M13 9PL, United Kingdom; 7 Department of Microbiology and Immunology, Indiana University School of Medicine, Indianapolis, USA; 8 Laboratory of Pharmaceutical Immunology, Faculty of Pharmaceutical Sciences, Tokyo University of Science, 2641 Yamazaki, Noda, Chiba, 278-8510, Japan; 9 School of Medicine, Trinity Translational Medicine Institute, Trinity College Dublin, Dublin, Ireland

**Keywords:** Innate lymphoid cells, ILC3, Bcl6, transcription factor, IL-17

## Abstract

Innate lymphoid cells (ILCs) are tissue-resident effector cells with roles in tissue homeostasis, protective immunity, and inflammatory disease. Group 3 ILCs (ILC3) are classically defined by the master transcription factor RORγt. However, ILC3 can be further subdivided into subsets that share type 3 effector modules that exhibit significant ontological, transcriptional, phenotypic, and functional heterogeneity. Notably lymphoid tissue inducer (LTi)-like ILC3 mediate effector functions not typically associated with other RORγt-expressing lymphocytes, suggesting additional transcription factors contribute to dictate ILC3 subset phenotypes. Here we identify Bcl6 as a subset-defining transcription factor of LTi-like ILC3 in mice and humans. Deletion of Bcl6 results in dysregulation of the LTi-like ILC3 transcriptional program and markedly enhances expression of IL-17A and IL-17F in LTi-like ILC3, in a manner in part dependent upon the commensal microbiota - and associated with worsened inflammation in a model of colitis. Together these findings redefine our understanding of ILC3 subset biology.

## Introduction

Innate lymphoid cells (ILCs) are a transcriptionally and biologically heterogenous group of tissue-resident effector cells with critical roles in homeostatic tissue function and protective immune circuits^1–3^. Current nomenclature for ILC subsets was informed by the overlapping expression of master transcription factors and effector cytokines with T helper cell subsets^4,5^. Accordingly, group 3 ILC (ILC3s) are considered an innate analogue of Th17 cells and are defined by the expression of RORγt, production of cytokines including interleukin (IL)-17A, IL-17F and IL-22 and roles in antimicrobial responses at barrier sites^1–4^. Nonetheless, ILC3s exhibit additional heterogeneity and in mice can be further divided into distinct subsets: lymphoid tissue-inducer (LTi)-like ILC3, natural cytotoxicity receptor-expressing (NCR^+^) ILC3 and “double negative” (DN) ILC3^2^. Importantly, these ILC3 subsets exhibit profound differences in their ontogeny, transcriptional signature, effector functions and tissue localization^2,6–8^. NCR^+^ ILC3 additionally produce the cytokine IFN-γ, exhibit non-classical cytotoxic function and can transdifferentiate to become ILC1-like upon loss of RORγt and in inflammatory settings^9,10^. The divergent functions of NCR^+^ ILC3 and DN ILC3 can be explained by the co-expression of T-bet, alongside RORγt, and have been elegantly dissected *in vitro* and *in vivo* in mouse and humans^9–11^.

In contrast, LTi-like ILC3 – defined by expression of CCR6, c-kit and Neuropilin-1 (Nrp1) in mice – are increasingly appreciated to undergo extensive immunomodulatory crosstalk with both innate and adaptive immune cells, and non-hematopoietic cells, via a range of mechanisms not typically observed in other RORγt-expressing lymphocyte populations^2,6^. Specifically, LTi-like ILC3 co-localize within tissue-associated lymphoid structures and draining lymph nodes where they act as antigen-presenting cells to modulate adaptive immune responses via MHCII and a number of auxiliary co-stimulatory molecules^6,12–17^. Moreover, we have previously demonstrated that RORγt is in part dispensable for the maintenance of LTi-like ILC3 phenotype and IL-22 production post-development^18,19^, further suggesting roles for additional transcription factors in imprinting and supporting the unique biological functions of this ILC3 subset. However, the transcription factors that shape LTi-like ILC3 function remain poorly understood.

In previous work, ourselves and others have described phenotypic and functional overlap in the biology of LTi-like ILC3 and adaptive immune cell subsets involved in humoral immunity. For example, both fetal LTi and LTi-like ILC3 in adults express CXCR5 and PD-1^20–23^ - phenotypes shared with T follicular helper cells (TfH)^24^. In addition, LTi-like ILC3, TfH and B cells utilize Gpr183 to position themselves within the interfollicular border of lymph nodes^13,25^, and intestinal-associated cryptopatches^17^. Moreover, we have previously demonstrated that the shared ability of these cells to respond to Gpr183-dependent migratory cues facilitates LTi-like ILC3 suppression of TfH responses and mucosal IgA responses in an MHCII-dependent manner^13^. Together, these findings provoked the hypothesis that LTi-like ILC3 could share transcriptional programs with adaptive immune cells involved in humoral immunity, which could explain aspects of the unique function and phenotype of this ILC3 subset.

Here we demonstrate that LTi-like ILC3 express the transcription factor Bcl6 in mice and humans. Deletion of Bcl6 in murine ILC3s led to alterations in the LTi-like ILC3 transcriptome, most notably a de-repression of the type 3 cytokines IL-17A and IL-17F in a manner partly dependent on the commensal microbiota and which was associated with exacerbated intestinal inflammation in a model of colitis. Together these findings identify Bcl6 as a subset-specific defining transcription factor of LTi-like ILC3 and expand the traditional analogy between ILC subsets and T helper cell subsets by implicating LTi-like ILC3 as a partial transcriptional innate counterpart of TfH.

## Results

### Bcl6 is expressed by murine intestinal LTi-like ILC3

**Figure 1:**
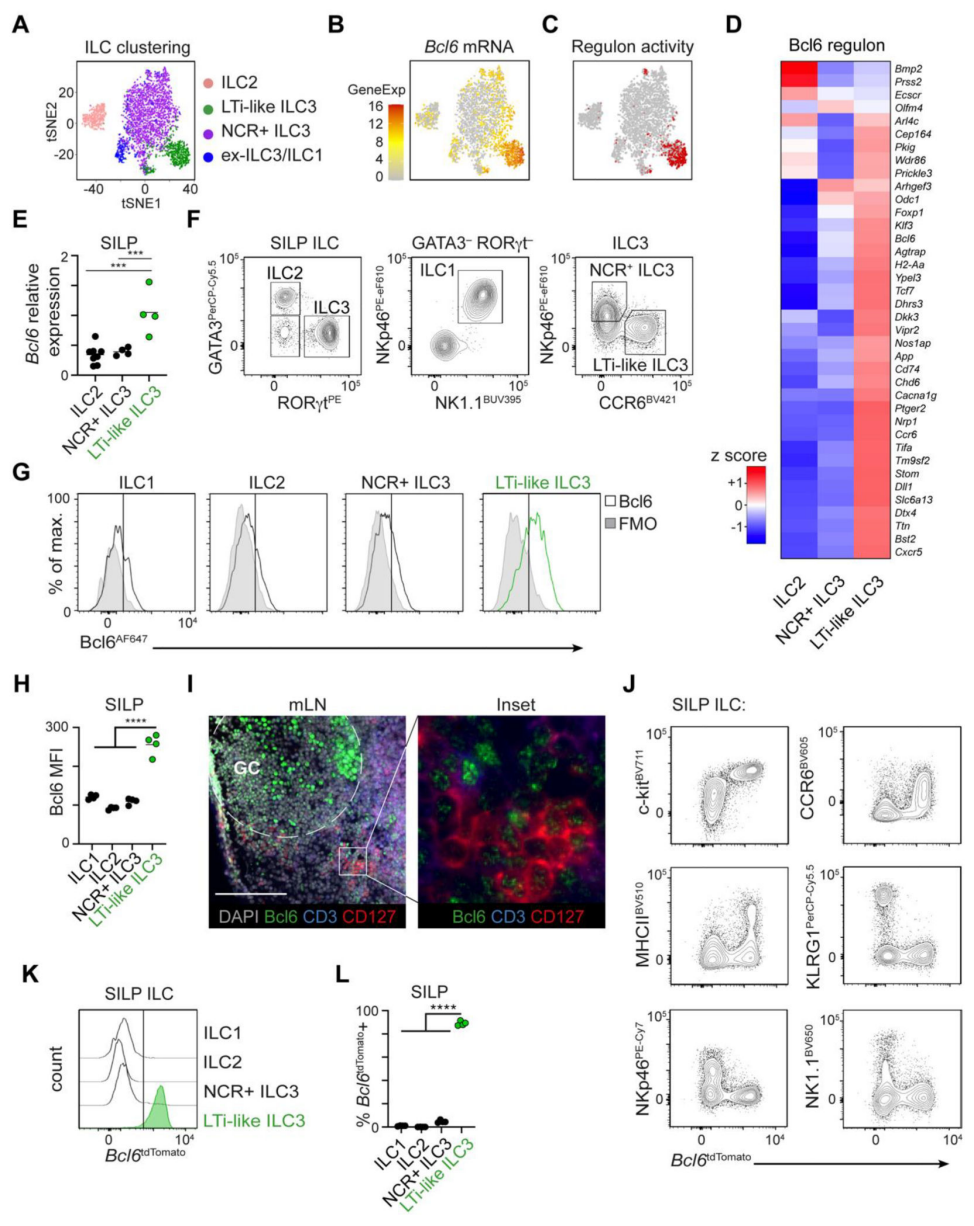
Bcl6 is expressed by murine LTi-like ILC3. A) t-SNE plot showing ILC superclusters, B) *Bcl6* mRNA expression and C) Bcl6 regulon activity in previously published scRNA seq analysis of ILC isolated from the small intestine lamina propria (SILP) of Id2^CreERT2^ mice ^19^. D) Heatmap showing relative expression of Bcl6 regulon-associated genes by ILC subsets from a publicly-available RNAseq data set (Immgen.org) ^43^. E) Relative expression of *Bcl6* mRNA on ILCs sorted from the SILP of RORγt^eGFP^ mice, determined by RT-PCR, n=4-8. F) Representative flow cytometry gating strategy used to identify ILC subsets from the total ILC population (CD45+, CD11b- CD11c- B220- CD3- CD5-, CD127+ CD90.2+) in murine SILP. G) Representative histograms, and H) geometric mean fluorescence intensity of Bcl6 protein across ILC subsets in mouse SILP, n=4. I) Immunofluorescence imaging of murine mLN stained for Bcl6 (green), CD3 (blue), CD127 (red) and DAPI (grey). Scale bar 100μm. J) Unbiased gating and expression of subset-defining surface markers versus Bcl6^tdTomato^ amongst total SILP ILCs (as in F), from Bcl6^tdTomato^ reporter mice. K) Representative histograms L) quantification of Bcl6^tdTomato^ expression amongst pre-gated ILC subsets from the SILP, n=4. Statistical significance was calculated using a one-way ANOVA (E, H, L) with Sidak’s (E) or Dunnet’s (H, L) post hoc test. Data pooled from two independent experiments (E), or representative of 2-3 independent experiments (F-L). Data represented as individual animals and mean. See also Figure S1 and Figure S2.

Utilizing prior single cell RNA sequencing (scRNA seq) data of small intestinal ILCs^19^ we dissected the transcriptional networks that could regulate ILC3 subset phenotype and function (Figure 1A). Further analysis of transcription factor mRNA expression, alongside predicted regulon activity, revealed Bcl6 as a transcription factor uniquely associated with ILCs expressing an LTi-like ILC3 signature (Figure 1B+C)^19^. LTi-like ILC3, but not NCR^+^ ILC3 or ILC2, were found to be enriched for the Bcl6 regulon-associated gene signature (Figure 1C), which included a number of genes previously reported to be selectively expressed by this subset including *Ccr6, H2-Aa, Cd74, Nrp1, Cxcr5* and *Odc1* (Figure 1D). To validate *Bcl6* expression at the mRNA level we sort-purified ILC subsets from the small intestinal lamina propria (SILP) or mesenteric lymph node (mLN) of RORγt^eGFP^ mice, and consistently observed enriched expression of *Bcl6* in LTi-like ILC3 in line with our scRNA seq data (Figure 1E, Fig S1A). Similarly, we could detect Bcl6 protein via flow cytometry in LTi-like ILC3 from the small intestine (Figure 1F-H) and mLN (Fig. S1B+C). Moreover, Bcl6 was similarly detected by immunofluorescence imaging in CD3^-^CD127^+^ ILCs localized at the interfollicular border of the mLN (Figure 1I), in line with our previous findings that LTi-like ILC3 inhabit this cellular niche to interact with adaptive immune cells^13,14^. To more comprehensively analyse *Bcl6* expression across tissues and within the ILC family we additionally utilized a Bcl6^tdTomato^ reporter mouse (Figure 1J-L). Unbiased analysis of Bcl6^tdTomato^ expression amongst Lineage^neg^ CD127^+^ CD90^+^ ILCs in the small intestine revealed reporter expression to be restricted predominantly to cells with a c-kit^+^ CCR6^+^ MHCII^+/-^ KLRG1^-^ NKp46^-^ NK1.1^-^ phenotype, further suggestive of restricted expression amongst LTi-like ILC3 (Figure 1J). In line with this, direct gating on LTi-like ILC3 revealed high expression of Bcl6^tdTomato^, which was absent in ILC1, ILC2 or NCR^+^ ILC3 in the small intestine (Figure 1K+L), mLN, colon and Peyer’s patches (Fig. S1D+E). Bcl6 is typically associated with a broader network of complementary and antagonistic transcriptional programs that act together to enforce cellular identity^24^. In line with this we found that while LTi-like ILC3 lacked expression of Blimp-1 (*Prdm1*) and T-bet, they highly expressed TCF-1 (*Tcf7*) when compared to other ILC subsets by both endogenous antibody staining and reporter allele expression (Fig. S1F-I).

Intriguingly, we observed Bcl6 expression to be absent in neonatal mice with expression arising by ~3 weeks post-birth (Fig. S2A-B), and persisting thereafter, suggesting Bcl6 is postnatally regulated. LTi-like ILC3 are subject to cues from the commensal microbiota and cell-cell interactions with adaptive immunity, thus we determined whether Bcl6 expression is dependent upon these contextual signals. Bcl6 expression was found to be comparable in LTi-like ILC3 from germ free, ex-germ free and SPF mice (Fig. S2C) - with germ free status confirmed via assessment of IL-17A+ CD4^+^ T cells (Fig. S2D) - as well as following antibiotic treatment of wild type SPF animals (Fig. S2E), suggesting the microbiota is not required for expression of this transcription factor. Similarly, Bcl6 was detected in LTi-like ILC3 from *Rag1*^-/-^ mice although intensity of staining was found to be slightly reduced from wild type counterparts (Fig. S2F). To determine whether Bcl6 expression was altered by cell activation, we assessed expression in LTi-like ILC3 from mice infected with *C. rodentium* as well as wild type cells stimulated *ex vivo* with IL-β and IL-23, but in both cases Bcl6 expression remained unchanged (Fig. S2G-H). Together these findings demonstrate Bcl6 to be a stable, postnatally acquired transcriptional program of LTi-like ILC3 in mice.

### Bcl6 expression is conserved in a subset of human ILC3

**Figure 2:**
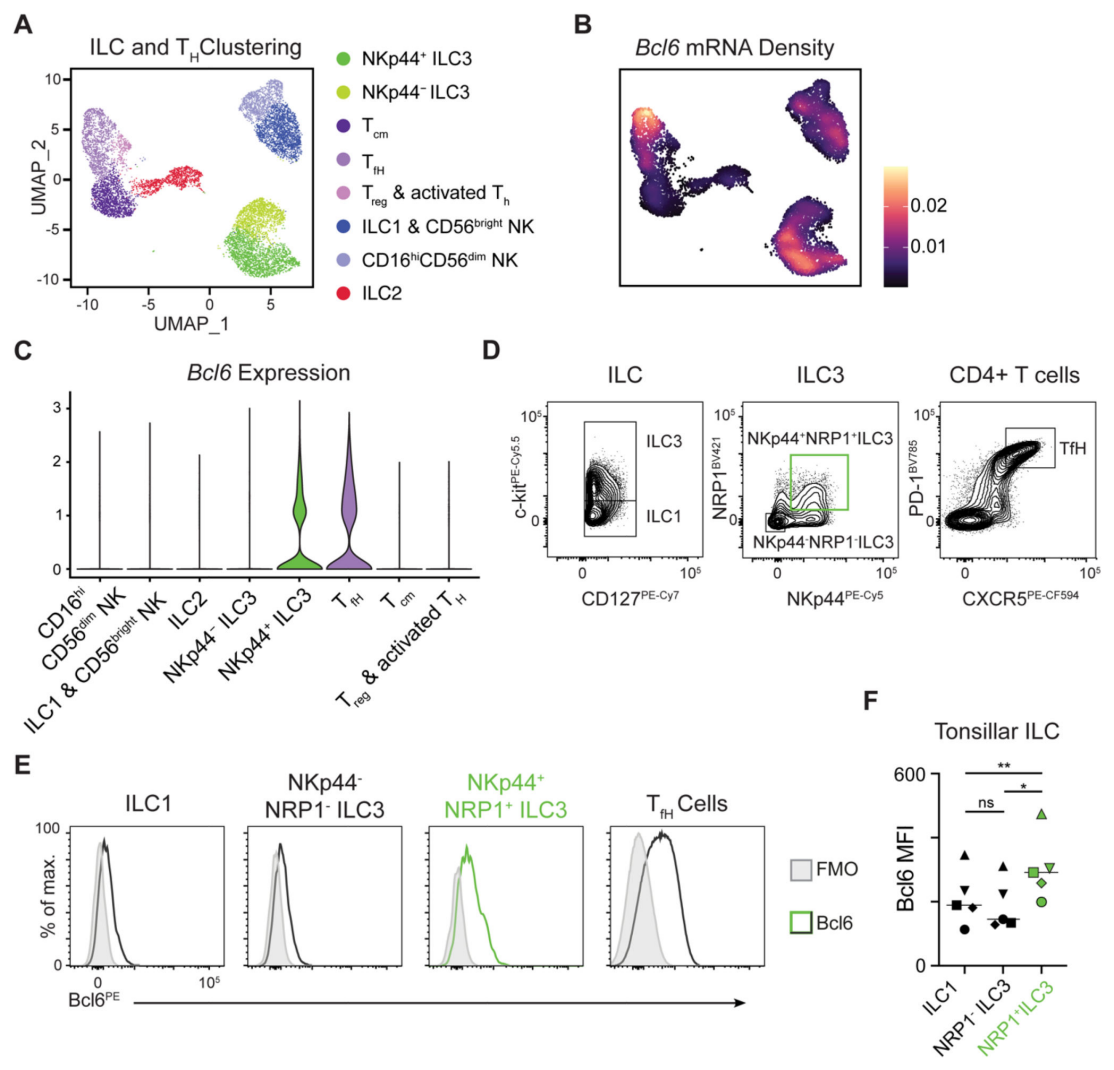
Human tonsillar Nrp1^+^ NKp44^+^ ILC3 express Bcl6. A) UMAP showing ILC and helper T cell clusters and B) *Bcl6* mRNA expression density from scRNA seq data generated from human tonsil cells. C) Violin plot summarizing *Bcl6* expression from cell clusters (as identified in A). D) Representative flow cytometry gating of ILC subsets (from CD45+ Lin- CD3- CD127+ CRTH2-), ILC3 subsets (from CD45+ Lin- CD3- CD127+ CRTH2- c-kit+) and TfH cells (from Lin- CD3+ CD4+) from the human tonsil. E) Representative histograms and F) geometric mean fluorescence intensity quantification of Bcl6 protein expression from ILC1, NKp44- NRP1- ILC3, NKp44+ NRP3+ ILC3 or control TfH cells from human tonsils, n=5. Statistical significance was determined by a one-way ANOVA with Tukey’s Multiple Comparison test (F). Bars on plot signify median. Data pooled from 2 independent experiments (A-C), or representative of 5 independent donors (D-F). See also Figure S3.

Next, we determined whether BCL6 expression was conserved in human ILC3s. Single cell sequencing of total lymphocytes isolated from human tonsils clearly distinguished ILC subsets, as well as heterogeneous populations of T cells (Figure 2A). As expected, *BCL6* expression was detected amongst tonsillar cells exhibiting a T follicular helper (TfH) cell signature (Figure 2B+C). In line with our findings in mice, *BCL6* transcript was enriched in ILC3s (Figure 2B+C) – in particular those exhibiting a phenotype (NKp44+) previously associated with mature, activated cells in humans^26^. To further investigate the transcriptional signatures associated with *BCL6* expression in ILC3 we isolated and reclustered ILC3 from the tonsil scRNA seq data set (Fig. S3). Analysis of isolated human tonsillar ILC3 revealed 5 subclusters (Fig. S3A). *BCL6* was found to be relatively broadly expressed and detected in 4 out of 5 ILC3 clusters (Fig. S3B-E). In particular *BCL6* was enriched amongst ILC3 expressing *NRP1* and *NCR2* (encoding NKp44) (Fig. S3C-D). The ILC3 subclusters identified exhibited differential expression of several genes (Fig. S3F), with *BCL6* expressing cell clusters also enriched for markers of activation and type 3 effector functions (Fig. S3F-G). ILC3 subclusters also exhibited a gradient in expression of transcription factors *AHR, BATF, TOX2* and *IKZF2* (Fig. S3H-I). Notably the lone ILC3 cluster lacking *BCL6* expressed *PRF1* (encoding Perforin) and higher expression of *SELL*, which has been reported to mark naive or immature ILC3 populations^27^ (Fig. S3J). We next investigated BCL6 at the protein level via flow cytometry – confirming signal in TfH as expected – and further validating expression of BCL6 as highest amongst NKp44^+^ NRP1^+^ ILC3, whereas NKp44^-^ NRP1^-^ ILC3 and ILC1 expressed relatively low levels of BCL6 across multiple individual donors (Figure 2D-F). Thus, our findings suggest BCL6 is a conserved transcriptional signature of human NKp44^+^ NRP1^+^ ILC3 – a phenotype analogous to lymphoid tissue-resident murine LTi-like ILC3^28^.

### Bcl6 shapes the transcriptional profile of LTi-like ILC3

**Figure 3:**
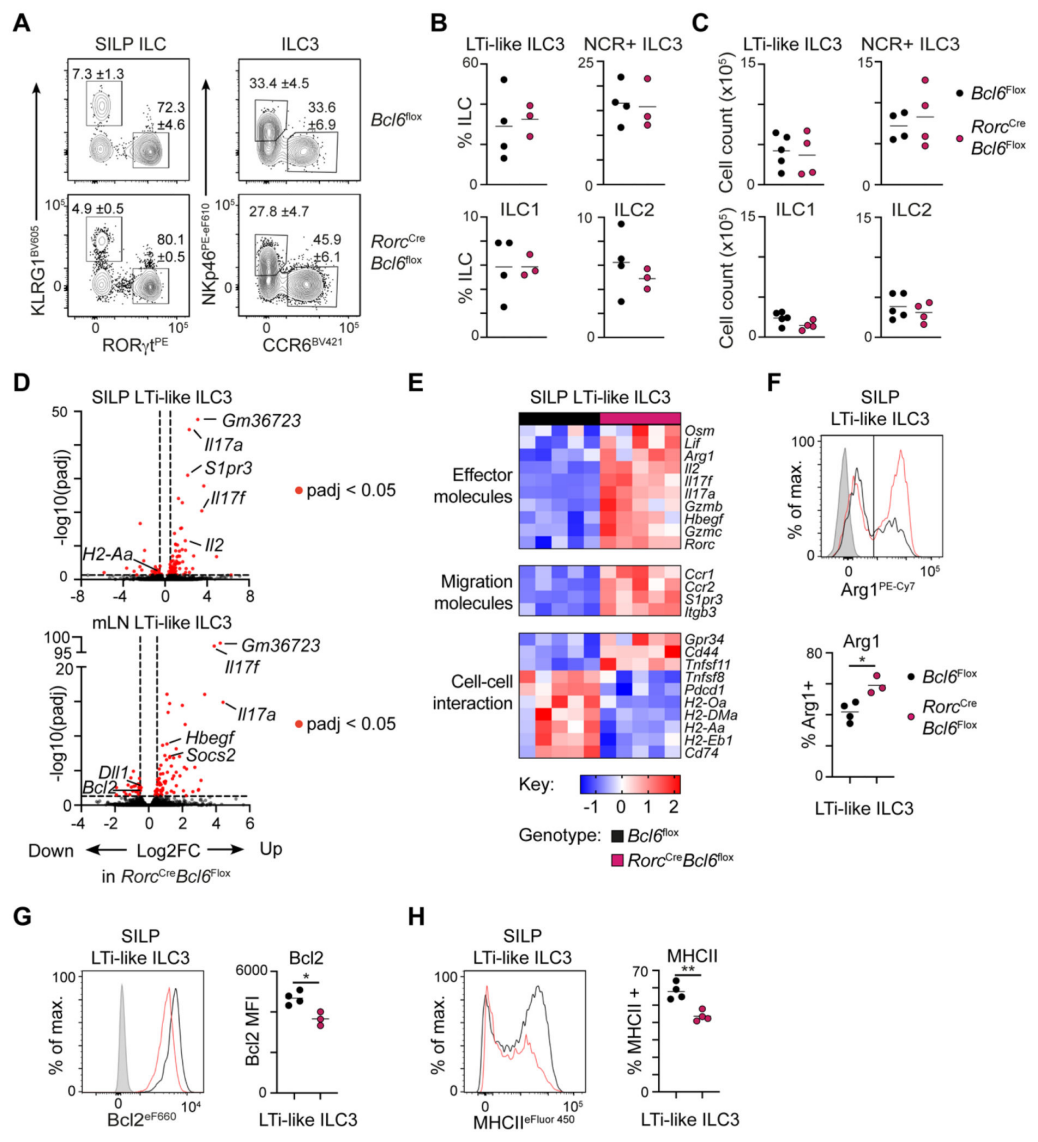
Bcl6 shapes the transcriptional landscape of LTi-like ILC3. A) Representative flow cytometry plots identifying ILC subsets from the total ILC population (CD45+, CD11b- CD11c- B220- CD3- CD5-, CD127+ CD90.2+). Values indicate average frequency (± standard error of the mean). B) Quantification of ILC subset frequency, n=3-4, C) and absolute cell counts in the SILP of *Rorc*^Cre^ x *Bcl6*^fl/fl^ and *Bcl6*^fl/fl^ control mice, n=4-5. D) Volcano plot summarizing gene fold change (Log2FC) and adjusted P value (-log10(padj)), between LTi-like ILC3 of *Rorc*^Cre^ x *Bcl6*^fl/fl^ and *Bcl6*^fl/fl^ control mice in the SILP (top), and mLN (bottom). E) Heatmap summarizing relative expression of curated and significantly differentially expressed genes between LTi-like ILC3 from the SILP of *Rorc*^Cre^ x *Bcl6*^fl/fl^ and *Bcl6*^fl/fl^ control mice. (F-H) Representative flow cytometry histograms and summary quantification plots of F) Arg1 G) Bcl2 and H) MHCII on LTi-like ILC3 from the SILP from *Rorc*^Cre^ x *Bcl6*^fl/fl^ and *Bcl6*^fl/fl^ control mice, n=3-4. Data representative of 3-5 independent experiments (A-C, F-H). Statistical significance was determined by an unpaired t-test (F-H). Data represented as individual animals and mean. See also Figure S4 and Figure S5.

To determine the role of Bcl6 in shaping ILC3 responses we generated conditional knockout mice (*Rorc*^Cre^ x *Bcl6*^fl/fl^) and validated deletion of the targeted exon in sort-purified LTi-like ILC3 (Fig. S4A). Deletion of *Bcl6* was found to be dispensable for normal development of LTi-like ILC3, and both frequencies and total numbers of LTi-like ILC3 and other major ILC subsets were unaltered in the small intestine (Figure 3A-C), colon and mLN (Fig. S4B) of *Rorc*^Cre^ x *Bcl6*^fl/fl^ mice. Lymphoid organogenesis was not disrupted by loss of *Bcl6* expression in LTi-like ILC3 as evident from conserved development and cellularity of mLN, Peyer’s patches and cryptopatches in *Rorc*^Cre^ x *Bcl6*^fl/fl^ mice (Fig. S4C-H). In line with these observations, levels of surface lymphotoxin heterotrimers on Bcl6-deficient LTi-like ILC3 remained intact (Fig. S4I). Thus, these data suggest Bcl6 is dispensable for the lymphoid tissue-inducer functions of ILC3.

To determine the role of Bcl6 in shaping the ILC3 transcriptome we sort-purified LTi-like ILC3 from the mLN and small intestine, together with small intestinal NCR^+^ ILC3, from littermate *Rorc*^Cre^ x *Bcl6*^fl/fl^ and Cre-negative *Bcl6*^fl/fl^ control mice and performed bulk RNAseq. Deletion of *Bcl6* led to marked changes to gene expression in LTi-like ILC3 from the mLN and small intestinal lamina propria (Figure 3D, Fig. S5A), whereas small intestinal NCR^+^ ILC3 exhibited relatively few differentially expressed genes (DEG) (Fig. S5B-C) – in agreement with our observation that Bcl6 is expressed primarily in the former ILC3 subset. In line with the role of Bcl6 as a potent transcriptional repressor^24^ the majority of DEG in LTi-like ILC3 from *Rorc*^Cre^ x *Bcl6*^fl/fl^ mice were found to be upregulated (Figure 3D), and encompassed a wide range of known effector cytokines, molecules involved in cell-cell interactions and phenotypic markers previously shown to have physiological relevance in LTi-like ILC3 function (Figure 3E). Nonetheless, a number of transcripts were also found to decrease in the absence of Bcl6 - including those involved in antigen presentation(Figure 3D, Fig. S5A). We further validated Bcl6-dependent changes at the protein level for a set of known markers including Arginase-1^29^ (Figure 3F), Bcl2^30^ (Figure 3G) and MHCII^15,16^ (Figure 3H), although the latter was found to be unaltered at the level of protein expression in the mLN (Fig. S5D). In line with conservation of MHCII on LTi-like ILC3 within lymphoid tissues, we did not observe changes in frequencies of Th17, TfH or germinal centre B cells (Fig. S5E-G), as previously observed following conditional deletion of ILC3-intrinsic MHCII^13,15,16^.

**Figure 4:**
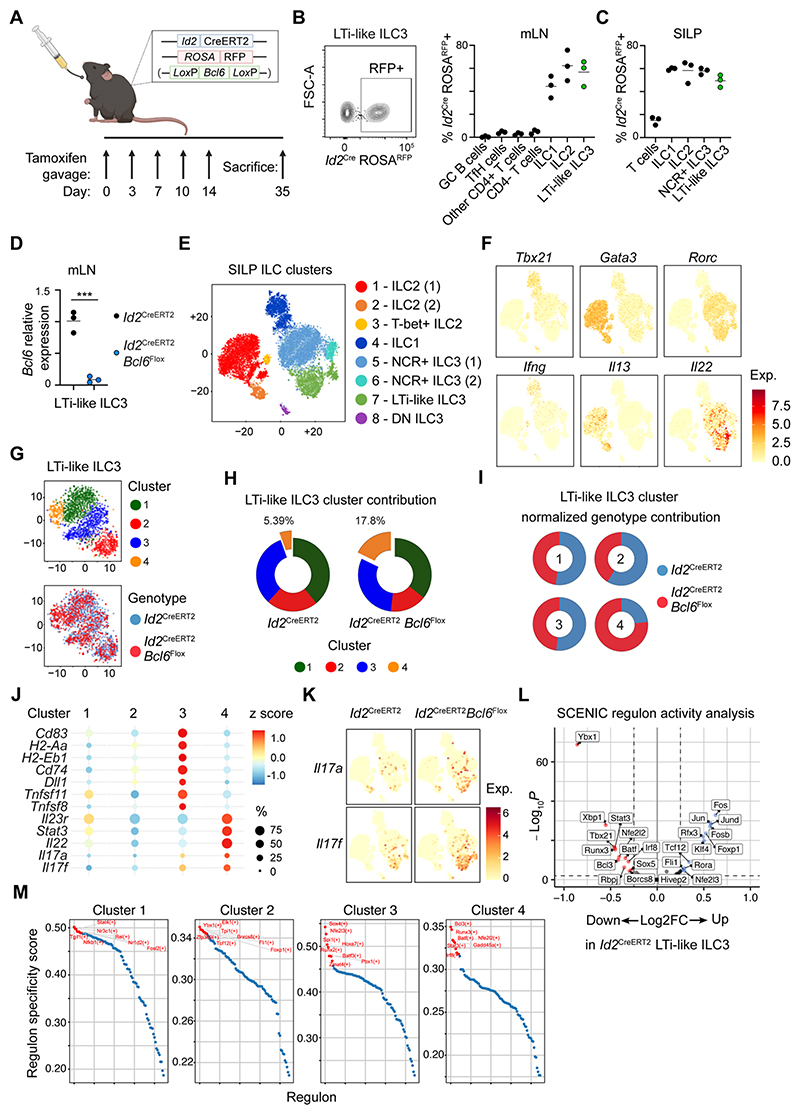
Single cell sequencing of inducible Bcl6-deletion in ILCs. A) Diagram of experimental design for Cre activation on *Id2*^CreERT2^ and *Id2*^CreERT2^ x *Bcl6*^fl/fl^ mice. B) Representative flow cytometry plot (left) and quantification (right) of *Id2*^CreERT2^ activation reflected by ROSA^tdRFP^ expression, on lymphocyte subsets from B) the mLN and C) SILP of *Id2*^CreERT2^ mice following tamoxifen administration, n=3. D) Relative *Bcl6* expression determined by RT-PCR on sort-purified tdRFP^+^ LTi-like ILC3 from the mLN of *Id2*^CreERT2^ and *Id2*^CreERT2^ x *Bcl6*^fl/fl^ mice, n=3. E) t-SNE and cluster identification of ILC single-cell RNAseq from the SILP of *Id2*^CreERT2^ and *Id2*^CreERT2^ x *Bcl6*^fl/fl^ mice. F) mRNA expression of subset-defining transcription factors and cytokines related to (E). G) LTi-like ILC3 sub-cluster identification (top) and genotype origin (bottom). H) Relative enrichment of LTi-like ILC3 sub-clusters on *Id2*^CreERT2^ and *Id2*^CreERT2^ x *Bcl6*^fl/fl^ mice, and I) relative genotype contribution to each LTi-like ILC3 sub-cluster as identified in (G). J) Dot-plot depicting frequency and normalised gene expression of selected genes across LTi-like ILC3 sub-clusters from (G). K) *Il17a* and *Il17f* mRNA expression on total ILCs from *Id2*^CreERT2^ and *Id2*^CreERT2^ x *Bcl6*^fl/fl^ mice, clustered as in (E). L) Volcano plot showing fold change (Log2FC) and adjusted P value (-log10(padj)) of regulon activity determined by SCENIC on LTi-like ILC3 from *Id2*^CreERT2^ or *Id2*^CreERT2^ x *Bcl6*^fl/fl^ mice. M) Regulon specificity score for each LTi-like ILC3 sub-cluster as identified in (G). Red labels indicate top most specific regulons in each cluster. Data representative of 2-5 independent experiments (B-D). Data represented as individual animals and mean. See also Figure S4 and Figure S6.

To further define intrinsic Bcl6-dependent effects on ILC gene expression we generated a second, tamoxifen-inducible model to target all ILCs. Previously described *Id2*^CreERT2^ combined with a ROSA26^flSTOP-tdRFP^ reporter allele^18,19^ were crossed with *Bcl6*^fl/fl^ mice (hereafter referred to *Id2*^CreERT2^ x *Bcl6*^fl/fl^), and deletion was induced in mature *Id2*-expressing ILCs via tamoxifen delivery to adult mice (Figure 4A-C). We confirmed deletion of Bcl6 in LTi-like ILC3 using this model (Figure 4D) and performed scRNA seq on total ILCs from the small intestine of these mice (Figure 4). Unbiased clustering revealed 8 distinct ILC clusters (Figure 4E), incorporating all major known ILC subsets (ILC1, ILC2, NCR^+^ ILC3, LTi-like ILC3) as defined by transcription factor and cytokine mRNA expression (Figure 4F). Deletion of Bcl6 was not sufficient to drive additional discrete clusters when comparing global ILC family signatures, indicating ILC subset-specific identity remained largely intact and acted as the major determinant of clustering at this level. Thus, we next isolated LTi-like ILC3 and re-clustered to reveal four sub-clusters – suggestive of transcriptional heterogeneity (Figure 4G). Analysis of LTi-like ILC3 sub-clusters by genotype revealed an enrichment of cells from *Id2*^CreERT2^ x *Bcl6*^fl/fl^ mice in sub-cluster 4 in comparison to wild type control cells (Figure 4H+I). Further analysis of gene signatures in sub-cluster 4 revealed a relative downregulation of genes associated with antigen-presentation and cell-cell interactions and an upregulation of effector cytokine genes (Figure 4J), mirroring our observations in bulk RNA seq analysis of LTi-like ILC3, and highlighted *Il17a* and *Il17f* as genes with increased expression in the absence of Bcl6 (Figure 4K and Figure 3D+E). To infer broader changes to LTi-like ILC3 transcriptional networks in the absence of Bcl6, we performed regulon analysis on the isolated LTi-like ILC3 subclusters, as previously described^19,31^. This analysis revealed wider changes to the transcriptional landscape of these cells (Figure 4L), with distinct enrichment of transcription factor regulons within individual sub-clusters (Figure 4M). Notably sub-cluster 4 – enriched in *Id2*^CreERT2^ x *Bcl6*^fl/fl^ mice – exhibited higher activity of the transcription factors Runx3, Batf and Stat3 amongst others (Figure 4M). Together these findings provoke the hypothesis that Bcl6 acts to modulate the transcriptional network within in LTi-like ILC3 and may act to repress type 3 cytokine production.

### Bcl6 antagonises type 3 cytokine production in LTi-like ILC3

ILC3s are classically characterised by a type 3 effector cytokine program. We previously demonstrated that mature tissue-resident ILC3s can maintain IL-22 production even in the absence of RORγt^18,19^. In contrast, loss of RORγt markedly reduced expression of IL-17A - indicating this cytokine as a primary target of this master transcription factor^19^. While inducible deletion of RORγt in this manner drives trans-differentiation of NCR^+^ ILC3 towards an NCR^+^ ex-ILC3/ILC1-like phentoype (Fig. S6A and as previously reported^19^), it did not alter Bcl6 expression within LTi-like ILC3 (Fig. S6B). Moreover, Bcl6 and its associated regulon remained undetected across all NCR^+^ ILC subsets even in the absence of RORγt, although intriguingly absence of this type 3 transcription factor modulated expression of some Bcl6 associated genes by LTi-like ILC3 (Fig. S6B+C), suggesting potential cross-regulation between these two master transcription factors.

**Figure 5:**
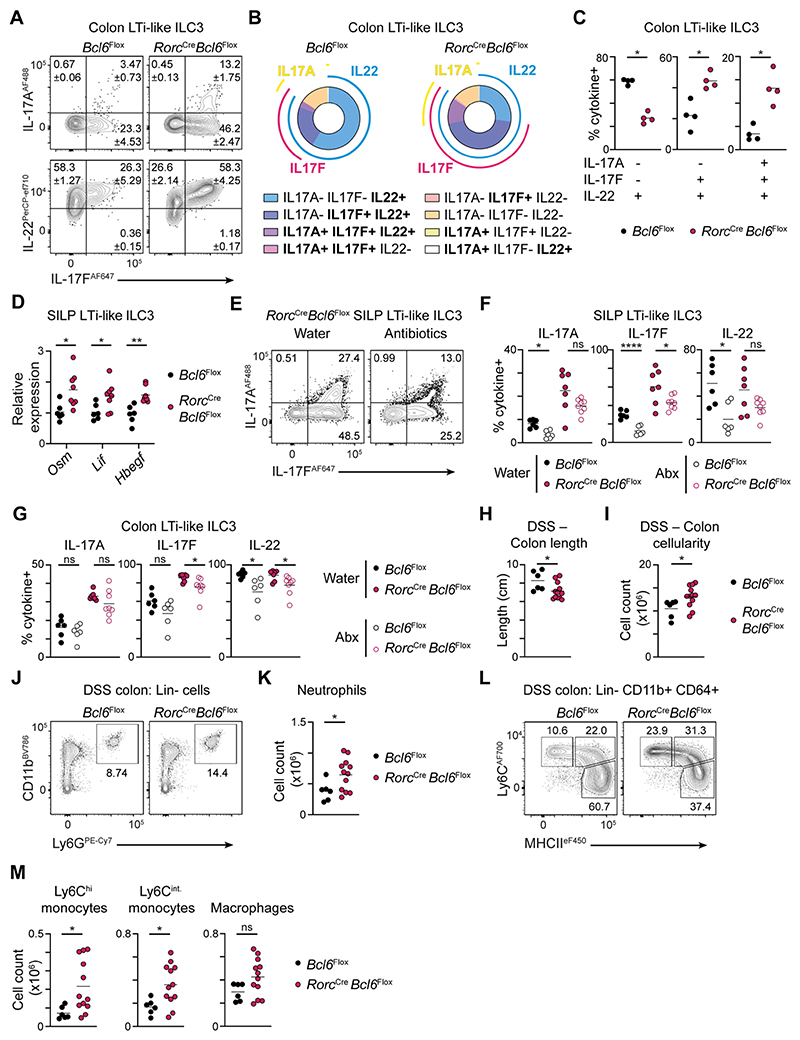
Bcl6 represses type 3 effector cytokine responses to the microbiota. A) Representative flow cytometry plots showing expression of IL-17A, IL-17F and IL-22 by LTi-like ILC3 from the colon of *Rorc*^Cre^ x *Bcl6*^fl/fl^ and *Bcl6*^fl/fl^ control mice. B) Pie charts depicting the average percentage of cells co-expressing one or more cytokine within LTi-like ILC3 from the colon of *Rorc*^Cre^ x *Bcl6*^fl/fl^ and *Bcl6*^fl/fl^ control mice. C) Quantification of single (IL-22+), double (IL-22+ IL-17F+) and triple (IL-22+ IL-17F+ IL-17A+)-secreting LTi-like ILC3 from the colon of *Rorc*^Cre^ x *Bcl6*^fl/fl^ and *Bcl6*^fl/fl^ control mice, n=4. D) Relative expression of *Osm, Lif*, and *Hbegf* mRNA on LTi-like ILC3 sorted from the SILP of *Rorc*^Cre^ x *Bcl6*^fl/fl^ and *Bcl6*^fl/fl^ control mice, determined by RT-PCR, n=6-8. E) Representative flow cytometry plots showing IL-17A and IL-17F production by LTi-like ILC3 from the SILP of water- or antibiotics-treated *Rorc*^Cre^ x *Bcl6*^fl/fl^ mice. (F-G) Quantification of frequencies of IL-17A, IL-17F and IL-22 producing LTi-like ILC3 from the F) SILP or G) the colon of antibiotics- or water-treated *Rorc*^Cre^ x *Bcl6*^fl/fl^ and *Bcl6*^fl/fl^ control mice, n=6-8. H) Length and I) absolute cell count of the colon of DSS-treated *Rorc*^Cre^ x *Bcl6*^fl/fl^ and *Bcl6*^fl/fl^ control mice, n=6-12. J) Representative gating strategy and K) neutrophil cell count in the colon, n=6-12. L) Representative gating strategies and M) cell counts of Ly6C^hi^ monocyte, Ly6C^int^ monocyte and macrophages in the colon, n=6-12. For myeloid cell analysis (J-M), cells expressing the lineage markers CD3, CD5, NK1.1 and CD19 were excluded. Data representative of 3-4 independent experiments (A-C), or pooled from 2 independent experiments (D-M). Statistical significance was determined using an unpaired Mann-Whitney test (C), unpaired t test (D, H-M), or an ordinary one-way ANOVA (F-G). Values on flow plots indicate average frequency (± standard error of the mean). Data represented as individual animals and mean. See also Figure S7.

Indeed, Bcl6 is known to largely act as a transcriptional repressor and has been reported to antagonise other lineage-associated transcription factors, including RORγt, as well as directly suppressing type 3 effector genes^24^. In line with our transcriptional data, we found that loss of LTi-like ILC3-intrinsic Bcl6 led to an enhanced capacity to produce both IL-17A and IL-17F in small intestinal and colonic LTi-like ILC3 (Figure 5A, Fig. S7A+B), but not NCR+ ILC3 (Fig. S7C+D). Notably we did not observe consistent changes in the total frequency of LTi-like ILC3 producing IL-22 (Fig. S7B, Figure 5B). However, we observed increased polyfunctionality amongst LTi-like ILC3 and increased frequencies of cells producing IL-22 along with either IL-17F alone, or with both IL-17A and IL-17F (Figure 5B+C). Similar observations were also made following inducible deletion of *Bcl6* (Fig. S7E+F). Additionally, we validated changes in the transcription of a wider signature of effector cytokines in Bcl6-deficient LTi-like ILC3, with higher levels of *Lif, Osm* and *Hbegf* (Figure 5D), but not *Csf2* (Fig. S7G). Moreover, we also observed a moderate but consistent increase in the intensity of RORγt expression in LTi-like ILC3 following *Bcl6* deletion (Fig. S7H) – suggestive of a potential role for Bcl6 in antagonising the RORγt program and thus, type 3 effector cytokine production.

Bcl6-dependent alterations in cytokine producing capacity were found to be more profoundly altered in the colon (Figure 5A-C, Fig. S7). Next, *Rorc*^Cre^ x *Bcl6*^fl/fl^ mice and control mice were challenged with the colonic pathogen *Citrobacter rodentium*. However, in line with a major role for IL-22 in *C. rodentium* clearance, we detected no significant change in bacterial burdens (Fig. S7I). Moreover, as at steady state, total numbers of LTi-like ILC3 were not altered in infected *Rorc*^Cre^ x *Bcl6*^fl/fl^ mice when compared to littermate controls (Fig. S7J). However, while IL-22 production was not affected, we detected an increase IL-17A^+^ cells in LTi-like ILC3 lacking Bcl6 (Fig. S7K).

We next hypothesised that Bcl6 may restrain IL-17A and IL-17F elicited by the commensal microbiota. To test this, *Rorc*^Cre^ x *Bcl6*^fl/fl^ mice and littermate control mice were administered a broad-spectrum cocktail of antibiotics, or water only, and found that antibiotic treatment was sufficient to partially reduce the production of IL-17A and IL-17F by Bcl6-deficient intestinal LTi-like ILC3 (Figure 5E-G). To determine whether elevated anti-commensal type 3 responses in *Rorc*^Cre^ x *Bcl6*^fl/fl^ mice led to changes in microbial composition we performed shotgun metagenomic analysis of fecal bacteria and observed moderate alterations in the composition of the commensal microbiota (Fig. S7L), that were largely driven by changes in the abundance of a limited number of species (Fig. S7M), although shifts in community composition at the genus level were relatively minor (Fig. S7N). Finally, to determine the consequences of elevated cytokine responses towards the microbiota in the context of Bcl6-deficiency, we exposed mice to dextran sulfate sodium (DSS)-induced colitis. *Rorc*^Cre^ x *Bcl6*^fl/fl^ mice exhibited an exacerbation of disease associated with worsened colon shortening (Figure 5H) and increased colonic cell numbers (Figure 5I) – the latter driven by increased numbers of infiltrating neutrophils and monocytes (Figure 5J-M). Together these data suggest Bcl6 acts to suppress type 3 effector cytokine responses to the commensal microbiota and limit inflammation upon loss of intestinal barrier integrity.

## Discussion

We have previously described biology shared by LTi-like ILC3 and the Bcl6-expressing arm of the adaptive immune response – including TfH and follicular B cells – which acts to facilitate regulatory crosstalk between these cell types^13^. Specifically, LTi-like ILC3 and Bcl6-expressing adaptive immune cells share surface phenotypes and utilize shared expression of migratory receptors to co-localize in lymphoid tissues. Building on prior evidence from our group^13^, and in line with a parallel study^32^, here we describe Bcl6 as a subset-defining transcription factor of LTi-like ILC3 in mice. We further demonstrate BCL6 expression is conserved amongst analogous NRP1^+^ ILC3 in the human tonsil, suggesting Bcl6 may be a conserved transcriptional signature of LTi-like ILC3 across species. Importantly, canonical ILC subset nomenclature was founded on analogous biology with T cell subsets^3–5^, however this paradigm has classically lacked ILC populations expressing master transcription factors of both regulatory T cells and TfH. Thus, these findings represent a paradigm shift and identify LTi-like ILC3 as the closest transcriptional and functional equivalent of Bcl6-expressing TfH.

In contrast, prior *in silico* and epigenetic analysis of ILC transcriptional networks had predicted Bcl6 as a transcriptional regulator of NK and ILC1^33^, provoking the need to clarify the expression of this transcription factor within the ILC family and its role in determining effector responses. Utilizing a combination of transcriptional and protein characterisation, together with reporter mice and conditional knockout models, our data identify Bcl6 as a transcription factor primarily associated with LTi-like ILC3. While RORγt has long been described as the master transcription factor of murine ILC3s, the transcriptional, phenotypic and functional heterogeneity evident in murine ILC3 subsets suggested likely roles for additional transcription factors in determining cell function. Our findings expand our understanding of the molecular underpinning of ILC3 subset heterogeneity, and suggest that similar to the imprinting of pro-inflammatory potential on NCR^+^ ILC3 by co-expression of RORγt and T-bet^9^, the phenotypes and functions of LTi-like ILC3 can be in part associated with co-expression of RORγt and Bcl6.

Simultaneous expression of both RORγt and Bcl6 in LTi-like ILC3 is somewhat surprising given Bcl6 has been attributed roles in suppressing other master transcription factor programs in T helper cells^24^, provoking the need to better understand the impact of concurrent transcription factor expression on the global network that determines cell identity and function in future studies. We also demonstrate high expression of TCF-1 in LTi-like ILC3 emphasising the activity of a broader Bcl6-associated program. Interestingly, a recent report demonstrates loss of TCF-1 in ILC3 results in a loss of Peyer’s patch formation^34^, which in contrast was not observed here in the absence of Bcl6. Conversely TCF-1 deletion was also associated with decreased levels of MHCII on small intestinal LTi-like ILC3, increased production of IL-17A and IL-17F, but normal levels of *Il22*^34^, phenocopying many of our findings here. These results suggest TCF-1 may act upstream of Bcl6 with both unique and overlapping functions. Moreover, all ILC family members express Id2 – another transcription factor classically antagonised by Bcl6^24^. These observations suggest a complex interplay between different transcriptional programs in LTi-like ILC3, which likely act as part of a broader network incorporating Id2^35^, RORα^19,35^, TCF-1^34,36^, BATF^37^ and Zbtb46^38^ to determine LTi-like ILC3 phenotype and function.

Bcl6 may act in part to antagonise the RORγt program and tune the magnitude of transcription of effector cytokines. Intriguingly we observed expression of Bcl6 only arose in LTi-like ILC3 post-birth at a time corresponding with weaning. Notably, one prior seminal study demonstrated IL-17A to be expressed robustly by LTi-like ILC3 in neonates immediately post-birth, but was significantly suppressed at 2 weeks of age and beyond^39^ – provoking the intriguing hypothesis that induction of Bcl6 expression in LTi-like ILC3 arises following birth to antagonise type 3 effector cytokine function and prevent inappropriate inflammation. The physiological consequences of this antagonism remain unclear but could feasibly act to prevent chronic production of IL-17A and IL-17F towards microbial stimuli to limit inflammation and help favour homeostatic IL-22 production and maintenance of barrier integrity. Interestingly, plasticity between RORγt-expressing Th17 and Bcl6-expressing TfH is also evident^40^, and critical in regulating IgA responses – another function shared with LTi-like ILC3^13,41^. Similarly, Bcl6 can bind RORγt target promoters to suppress type 3 cytokines in TfH ^42^, suggesting overlapping transcriptional regulation of mucosal immune effector functions in both innate and adaptive lymphocytes.

Our findings both complement and contrast a parallel publication reporting expression of Bcl6 by LTi-like ILC3^32^. In line with our data, both studies suggest that Bcl6 acts to repress several genes classically associated with RORγt, most notably IL-17. However, unlike the study by Li *et al*.^*32*^, we did not observe differences in expression of IL-22 and found IL-22-dependent immunity to *C. rodentium* infection to be intact in mice with Bcl6-deficient ILC3s. Moreover, we were unable to find evidence to support plasticity of LTi-like ILC3 or enhanced frequencies and numbers of ILC1 following intrinsic deletion of *Bcl6* using a combination of both constitutive *Rorc*^Cre^ or inducible *Id2*^ERT2Cre^ targeting, with proportions of major ILC subsets comparable in the absence of Bcl6. Indeed, many of the findings reported by Li *et al*^*32*^ utilized a *Vav1*^Cre^ based approach which has the potential to lead to confounding effects on other arms of the immune system and alter ILC3 responses indirectly, independent of any intrinsic effects of Bcl6 deletion. In addition, the authors suggested a role for the microbiota in the regulation of Bcl6 expression in LTi-like ILC3, as evidenced by a reduced frequency of Bcl6^+^ ILCs amongst total ILCs in germ free animals^32^. In contrast, we did not observe any reduction in cell-intrinsic expression of Bcl6 by LTi-like ILC3 in germ free or antibiotic treated animals in our hands. Nonetheless, we demonstrate depletion of the host commensal microbiota can in part reduce the elevated type 3 cytokine production observed following deletion of Bcl6 in LTi-like ILC3. This indicates the commensal microbiota is an important environmental cue in driving ILC3 effector responses, which are subject to Bcl6-dependent modulation, but not for Bcl6 expression *per se*. Finally, our work complements and extends these overlapping findings by demonstrating Bcl6 to be a conserved transcription factor signature of human NRP1+ ILC3 – a population thought analogous to LTi-like ILC3 in mice^28^.

Together these findings expand our understanding of the transcriptional networks that underpin phenotypic and functional heterogeneity between ILC3 subsets in mice and humans and implicate Bcl6 as a transcriptional regulator of homeostatic type 3 effector responses and host-microbial interactions in the intestinal tract.

### Limitations of the study

A key limitation of the current study is that the precise molecular mechanism through which Bcl6 regulates the transcriptional programming of LTi-like ILC3 remains unclear. Our data provoke the hypothesis that Bcl6 may act in part via repression of RORγt and its target genes, while Bcl6 is also known to antagonise other ILC-associated transcription factors including Id2 and RORα. Our bioinformatic analysis of both murine and human scRNA seq data revealed potential links between Bcl6 and other transcription factors in LTi-like ILC3 that will no doubt inform future studies but remain incompletely defined in this current study. Bcl6 mediates its functions largely as a “repressor-of-repressors” and the elucidation of its complex transcriptional network in other Bcl6-expressing cells, such as TfH, has taken significant effort to unravel^24^. Currently, technical challenges in performing targeted transcriptional and epigenetic studies (e.g. CHIP-Seq) have limited our ability to directly assess how Bcl6 functions in ILC and further studies will be needed to elucidate its in the context of the broader transcriptional network in LTi-like ILC3. An additional limitation of this current study is that we were unable to definitively address the consequences of Bcl6-deletion on the ability of LTi-like ILC3 to interact with, and modulate, TfH and the humoral immune response due to the lack of suitable mouse models to fully restrict deletion to the ILC lineage alone. The development of new tools and models to definitively target ILC in the presence of an intact adaptive immune system will allow for future studies to address this important question. Finally, we observed Bcl6 expression in LTi-like ILC3 arose at time points corresponding with weaning, however Bcl6 expression was not dependent upon microbial colonization. Future studies are needed to dissect the molecular cues and events that induce this transcription factor in the ILC family.

## STAR Methods

### Resource Availability

#### Lead Contact

Further information and requests for resources and reagents should be directed to and will be fulfilled by the lead contact, Matthew Hepworth (matthew.hepworth@manchester.ac.uk).

#### Materials Availability

This study did not generate new unique reagents.

### Experimental model and study participant details

#### Mice

Mice aged between 6-12 weeks were housed in individually ventilated cages at the University of Manchester, under specific pathogen free conditions, standard chow and water provided *ad libitum*, constant temperature and a 12 hour light and dark cycle. Female C57BL/6 mice were purchased from Envigo. Germ Free C57BL/6 and *Rag1*^-/-^ animals were bred and maintained at the University of Manchester. *RORc*^Cre^ and RORγt^eGFP^ mice were originally a kind gift from Gerard Eberl (Pasteur Institute, Paris). Bcl6^fl/fl^ were kindly provided by Alexander Dent (Indiana University School of Medicine). Bcl6^tdTomato-CreERT2^ mice ^44^, were kindly provided by Yohsuke Harada (Tokyo University of Science), and maintained at the University of Birmingham. *Id2*^CreERT2^ mice (Stock 016222), *Tcf7*^eGFP^ flox mice (B6(cg)-*Tcf7*^tm1Hhx^/J; Stock 030909) and *Rorc*^Flox/Flox^ mice (Stock 008771) were originally purchased from Jackson laboratories. ROSA-26^tdRFP^ mice were a kind gift from Hans-Joerg Fehling (University Clinics, Ulm, Germany). For transgenic mouse studies littermates of the same sex were assigned to experimental groups according to genotype. Where necessary or appropriate both female and male mice were utilized and sex was not found to be a confounding factor in the observations reported herein. To elicit Id2^CreERT2^ activation, tamoxifen was administered via oral gavage in 5 doses of 5 mg in 100 μl of corn oil, given throughout 14 days. All animal studies were performed under license of the U.K. Home Office and under approved protocols, carried out in accordance with the Animals (Scientific Procedures) Act 1986.

#### Human subjects

Tonsil tissue was obtained from seven patients undergoing tonsillectomy; five at Sophiahemmet Hospital, Sweden and two at Karolinska University Hospital, Sweden. The patients were two females aged 28 and 63 years old, and five males ages 3, 4, 5, 17 and 28 years old.

### Method Details

#### *Citrobacter rodentium* infection

Mice were orally gavaged with 200 μl containing 2–3 × 10^9^ colony forming units of *Citrobacter rodentium*. To produce the inoculum, Nalidixic acid-resistant *C. rodentium* ICC169 from a frozen glycerol stock was streaked in an LB agar plate containing 50 μg/mL nalidixic acid and grown overnight at 37 °C. Single colonies were then seeded into 15 ml of LB broth supplemented with 50 μg/mL nalidixic acid (Sigma-Aldrich) and grown for 18 h at 37 °C in agitation. Cultures were centrifuged, resuspended in 1.5 ml sterile PBS and used for gavage immediately. Mice were weighed daily, and fecal colony forming units were determined at day 5 and 6 post-infection. Briefly, fecal pellets were homogenized in sterile 10 μl PBS for each mg of feces and serial dilutions were plated LB agar plates containing 50 μg/mL nalidixic acid. Mice with no detectable colony forming units at day 5 were excluded from the experiment.

#### DSS-induced colitis

Mice were given drinking water containing 2.5 % dextran sulfate sodium (DSS; MP Biomedicals) for 4 days and returned to water only for 3 days prior to euthanasia.

#### Antibiotic treatment for microbiota depletion

Mice were orally gavaged with 200 μl drinking water containing 12.5 mg/ml of Ampicillin, Neomycin, Metronidazole and Gentamycin, and 6.25 mg/ml of Vancomycin. 7 doses were administered in the course of 14 days. Control mice were gavaged with drinking water.

#### Murine lymphocyte isolation

Lymph nodes and Peyer’s Patches, liver and thymus were collected in 1 ml of complete RPMI, mechanically disrupted and filtered through a 70 μm nylon strainer. For small and large intestine lamina propria lymphocyte isolation, Peyer’s Patches and fat were removed and intestines were opened longitudinally and shaken vigorously in PBS. Then, the mucus layer and epithelial cells were stripped by incubating tissue in PBS containing 1 mM EDTA, 1 mM dithiothreitol and 5 % FBS for 25 min at 37 °C. Then, tissues were washed in PBS and digested in complete RPMI with 0.1 mg/ml collagenase/dispase (Roche) and 20 μg/ml DNase (Sigma-Aldrich) for 40 min at 37 °C. Neonatal small intestine was cut into 3 mm pieces and digested in complete RPMI containing 0.5 mg/mL DNase I (Sigma-Aldrich) and 0.385 mg/m Liberase TM (Roche) for 40 min at 37 ºC. For small intestine lamina propria cell sorting, tissue was digested in 1 mg/ml collagenase VIII (Sigma-Aldrich) and 20 μg/ml DNase (Sigma-Aldrich) for 25 min at 37 °C. All digested tissues were filtered through a 70 μm nylon strainer prior to downstream use.

#### Human lymphocyte isolation

Freshly resected tonsil tissue was washed in PBS. Any visible signs of pus, blood, or burns were cut away and then tissue was further cut into smaller pieces before being mechanically disrupted by grinding and filtering. The filtered cell suspension was washed in 50 ml PBS, pelleted by centrifugation, resuspended in 20 ml PBS and layered onto 30 ml Ficoll (Cytiva) and centrifuged. The lymphocyte layer was harvested and washed with 50 m PBS before being pelleted by centrifugation and resuspended in 50 ml PBS. Cells were then counted using a Countess II (Invitrogen). After counting, 500 million cells were stained with Fc Block (Miltenyi Biotec) for 15 minutes, washed, and then stained with MACS anti-CD19 magnetic beads (Miltenyi Biotec) according to manufacturer’s instructions. After staining cells were washed and B cells were depleted using MACS LD columns (Miltenyi Biotec) according to manufacturer’s instructions. Remaining cells were counted using a Countess II (Invitrogen) and then used for staining for either flow cytometric analysis or for fluorescence-activated cell sorting (FACS).

#### *Ex vivo* cell stimulation

For the assessment of cytokine production *ex vivo*, 2 – 6 × 10^6^ cells were incubated in round bottom 96-well plates with complete RPMI supplemented with 20 ng/mL recombinant mouse IL-23 (Invitrogen) and 20 ng/mL recombinant mouse IL-β (Invitrogen) for 2 h. Then, eBioscience Cell Stimulation plus Protein Transport Inhibitor Cocktail (Invitrogen) were incorporated to the media at a final concentration of 2 ng/ml for another 3 h, for a total of 5 h. For the evaluation of Bcl6 levels after cytokine stimulation *ex vivo*, 2 – 6 × 10^6^ cells were incubated in round bottom 96-well plates with complete RPMI supplemented with 20 ng/mL recombinant mouse IL-23 (Life Technologies) and 20 ng/mL recombinant mouse IL-β (Life Technologies) for 4 h at 37 ºC. Cells were immediately used for downstream flow cytometry.

#### Mouse flow cytometry and cell sorting

Single cell suspensions were incubated in PBS containing 2 % FBS, 1 mM EDTA, and antibodies to the corresponding extracellular antigens for 30 min at 4°C. For LTα1β2 detection, cells were blocked with 40 mg/ml AffiniPure Fab Donkey Anti-Mouse IgG (Jackson Immunoresearch), and stained with Recombinant Mouse Lymphotoxin beta R/TNFRSF3 Fc Chimera (R&D Systems). Then, signal was amplified using Donkey Anti-Mouse IgG Biotinylated Antibody (R&D Systems), and detected using Streptavidin APC (eBioscience). For intracellular staining, cells were fixed and permeabilised using the FoxP3 Transcription Factor Buffer set (eBiosciences), or the BD Cytofix/Cytoperm buffer (BD Biosciences) for samples containing reporter proteins. Cells were then permeabilized using the FoxP3 Transcription Factor Buffer set (eBiosciences) and stained for intracellular antibodies. Flow cytometric data were collected using a BD LSRFortessa analyzer (BD Biosciences) using FACSDiva 8.0.2 software (BD), with data subsequently analyzed with FlowJo (version 10, Tree Star). For sorting, stained cells were sort-purified using FACSAria II (BD Biosciences). For all experiments, single leukocytes were identified based on forward and side scatter parameters, and expression of CD45. Dead cells were excluded using LIVE/DEAD Fixable Stain (Life Technologies). Total ILCs were gated based on lack of lineage markers (B220, CD11b, CD11c, CD3, CD5, +/- NK1.1) and expression of CD90.2 and CD127. Cells expressing Ter119, γδTCR and βTCR were further excluded for single-cell RNA seq cell sorting.

#### Human Cell Flow Cytometry

Freshly isolated cells were stained on the same day as the isolation. For staining, cells were incubated with LIVE/DEAD™ Fixable Green Dead Cell Stain Kit (Fisher Scientific) and surface protein antibodies at 4 °C for 30 min. Stained cells were washed with FACS buffer and resuspended in Foxp3 Fixation/Permeabilization buffer (eBiosciences) according to manufacturer’s instructions. Fixed cells were stained for Bcl6 with PE anti-Bcl-6 (BD Biosciences) at room temperature for 30 mins. Cells were washed twice with 2 mL 1x permeabilization buffer before being resuspended in FACS buffer. Flow cytometric data were collected using a BD LSR Fortessa analyzer (BD Biosciences) with FACS Diva version 8.01 software. Data was subsequently analysed with FlowJo (version 10, Tree Star). Single leukocytes were identified based on forward and side scatter parameters, and expression of CD45. Dead and lineage positive cells were excluded using LIVE/DEAD™ Fixable Green Dead Cell Stain Kit (Fisher Scientific) and lineage markers (CD1a, CD14, CD34, CD94, BDCA2, FcεRIα, CD123, TCRγδ, CD19). T helped cells were gated at CD45^+^CD3^+^CD4^+^ before further gating as CXCR5^+^PD1^+^ TfH cells. ILCs were gated as Lin^-^CD3^-^CD127^+^ before further gating into CD117^-^CRTH2^-^ ILC1s and CD117^+^CRTH2^-^ ILC3s, ILC3s were then gated as NKp44^+^NRP1^+^ or NKp44^-^NRP1^-^.

#### Human cell sorting

Freshly isolated cells were sorted on the same day as the isolation. For sorting, cells were incubated with LIVE/DEAD™ Fixable Near-IR Dead Cell Stain Kit (Fisher Scientific) and surface protein antibodies at 4 °C for 30 min, washed, and resuspended in FACS buffer (PBS with 2% FBS). Dead cells were excluded using LIVE/DEAD Fixable Near-IR Dead Cell Stain (Fisher Scientific) and lineage positive cells were excluded using lineage markers (CD1a, CD14, CD34, BDCA2, FcεRIα, CD123, TCRαβ, TCRγδ). T cells were sorted as CD45^+^CD3^+^, ILCs and NK cells were sorted into a single tube as CD45^+^Lin^-^CD3^-^CD19^-^NKG2A^-^CD56^-^CD127^+^, and CD45^+^Lin^-^CD127^-^CD56^+^, respectively. Cells were sorted into Eppendorf tubes containing Sort Buffer (PBS with 0.2% BSA and 5mM EDTA). Cells were sorted on a FACSAria III sorter (BD Biosciences), with FACS Diva version 9 software.

#### Immunofluorescence staining

Mesenteric lymph nodes were snap-frozen in OCT embedding matrix (Pfm Medical UK), cut into 7 μm sections and mounted into Superfrost Plus slides (VWR). Sections were fixed in cold acetone for 20 min and hydrated in PBS for 5 min. Then, slides were blocked with 10% horse serum in staining buffer (1% BSA in PBS). Slides were then stained with Alexa Fluor 488 Mouse anti-Bcl-6 (BD Biosciences) and biotinylated anti-mouse CD3e (eBioscience) in staining buffer for 1 h at room temperature. Slides were washed with PBS for 10 min and incubated with Alexa Fluor 488 Polyclonal Antibody (Thermo Fisher Scientific) secondary antibody for 30 min, for Bcl6 signal amplification. After washing, slides were incubated with goat anti-Rabbit IgG (H+L) FITC (Southern Biotech) tertiary antibody for 30min. After washing, slides were blocked with 10% rat serum in staining buffer for 30 min. Without washing, slides were incubated with CD127 Monoclonal Antibody (A7R34), eFluor™ 660 (Thermo Fisher Scientific) and Fluorescein/Oregon Green Polyclonal Antibody, Alexa Fluor™ 488 (Thermo Fisher Scientific) quaternary antibody for 30 min. After washing, slides were incubated with Donkey anti-Rabbit IgG (H+L) Alexa Fluor™ 488 (Thermo Fisher Scientific) quinary antibody and Streptavidin Alexa Fluor® 555 Conjugate (Thermo Fisher Scientific) for 30 min. For detection of RORγt, slides were stained with anti-RORγt antibody (eBioscience). Signal was amplified with FITC Donkey Anti-Rat IgG secondary antibody (Jackson Immunoresearch), Fluorescein/Oregon Green Polyclonal Antibody, Alexa Fluor™ 488 tertiary antibody (Thermo Fisher Scientific) and Donkey anti-Rabbit IgG (H+L) Alexa Fluor™ 488 quaternary antibody (Thermo Fisher Scientific). Secondary, tertiary, quaternary and quinary antibodies were cross-adsorbed for 30min in 10 % mouse serum in staining buffer before staining. Sections were counterstained with DAPI (Invitrogen) and mounted using ProLong™ Gold Antifade reagent (Thermo Fisher Scientific). Slides were visualized under a Zeiss Axio Imager.D2 fluorescence microscope. Images were processed with ImageJ.

#### RT-PCR

RNA was isolated from sorted cells using the Single Cell RNA Purification Kit (Norgen) and immediately transcribed into cDNA using the High-capacity cDNA reverse transcription kit (Applied Biosystems). Taqman Gene Expression assays or SybrGreen assays were used to determine gene expression. Data were normalised to the house-keeping gene *Hprt*.

#### Bulk RNA seq

RNA was isolated from sorted cells using the Single Cell RNA Purification Kit (Norgen) and amplified using SMART-Seq v4 Ultra Low Input RNA Kit for Sequencing (Takara Bio USA, Inc., Mountain View, USA). AMPure XP beads were used to purify the resulting ds-cDNA, which was then quantified using Qubit (Life Technologies). Libraries were prepared using NEBNext Ultra RNA Library Prep Kit (New England Biolabs, Ipswich, USA) following the manufacturer’s recommendations, and sequenced on Illumina NovaSeq 6000 S4 flow cell with PE150 according to results from library quality control and expected data volume. CASAVA based recognition was used to convert raw reads into FASTQ files. Reads with adapter contamination, reads with >10 % of uncertain nucleotides and reads with base quality <5 in over 50% of the read were filtered out. The remaining reads were aligned with HISAT2. Differential expression analysis was performed with “DESeq2” ^45^ on R v4.2.0 through RStudio v2022.07.01 (https://www.R-project.org/). Genes with <100 added counts across samples were filtered out. Genes were considered differentially expressed when adjusted p values were <0.05. Heatmaps of DEGs were generated with ”heatmap.2” function from “gplots” R package ^46^. Heatmap showing expression of genes included in the Bcl6 regulon across ILC subsets was performed using publicly-available ILC bulk RNAseq dataset GSE109125 ^43^.

#### Mouse single-cell RNA seq

Total ILCs were sorted as above into tubes containing PBS 10% FBS. Single-cell gene expression libraries were prepared using the Chromium Controller and Single Cell 3’ Reagent kits v3.1 (10x Genomics, In. Pleasanton, USA) as indicated on the manufacturer’s protocol (CG000315 Rev B). Briefly, nanofilter-scale Gel Beads-in-emulsion (GEMs) were generated by combining barcoded Gel Beads, cell-containing mastermix, and partitioning oil onto a Chromium chip. Cells were delivered at a limiting dilution to ensure that the majority (>90%) of GEMs contain no cell, while the reminder largely contain a single cell. The Gel Beads were then dissolved, primers released, and any co-partitioned cells lysed. Primers containing Illumina TruSeq Read 1 sequencing primer, a 10-nucleotide unique molecular identifier (UMI), a 16-nucleotide 10x Barcode, and a 30-nucleotide poly(dT) sequence were then mixed with the cell lysate and master mix containing reverse transcription reagents. Following GEM incubation, GEMs were broken and pooled fractions, containing barcoded cDNA from poly-adenylated mRNA, were recovered. First-strand cDNA was then purified from the post GEM-RT reaction mixture using silane magnetic beads and amplified via PCR. Enzymatic fragmentation and size selection were then used to optimize the cDNA amplicon size. Sample index, Illumina P5 and P7 sequences, and TruSeq Read 2 sequence were added via end repair, A-tailing adaptor ligation and PCR to yield final Illumina-compatible sequencing libraries. The resulting libraries comprised standard Illumina paired-end constructs flanked with P5 and P7 sequences. Read 1 contained 16 base-pair 10x Barcodes and 10 base-pair unique molecular identifiers (UMIs), while read 2 was used to sequence the cDNA. The i7 index read incorporated the sample index sequences. Paired-end sequencing (26:98) was performed on the Illumina NextSeq 500 instrument using NextSeq 500/550 High Output v2.5 (150 cycles) reagents.

#### Mouse single-cell RNA seq raw data processing and cell filtering

The 10x Genomics Cell Ranger pipeline (v6.1.2) was used to process raw sequencing data, whereby the .bcl files were demultiplexed and converted to FASTQ files using “cellranger mkfastq”. The FASTQ files were then mapped against the pre-built Mouse reference package from 10X Genomics (mm10-2020-A) using “cellranger count” to generate the gene-cell barcode matrix. For each sample, the single-cell data were processed in R environment (v4.1) following the workflow documented previously ^47^. Briefly, the HDF5 file generated by Cell Ranger was imported into R to create a SingleCellExperiment object. We used a combination of median absolute deviation (MAD), as implemented by the “isOutlier” function in the scuttle R package (v1.4.0) and exacted thresholds to identify and subsequently remove low quality cells before further processing. After filtering, 11708 cells (5369 from Id2^CreERT2^ ROSA-26^tdRFP^ and 6339 from Id2^CreERT2^ ROSA-26^tdRFP^ Bcl6^flox/flox^ mice) of 14100 cells remained for downstream analysis.

#### Mouse single-cell RNA-seq data integration, visualization and cell clustering

The “multiBatchNorm” function from the batchelor R package (v1.10.0) was used to re-compute the log-normalised expression values of the combined single-cell data. The per-gene variance of the log-expression profile was modelled using the “modelGeneVarByPoisson” function from the scran R package (v1.22.1). Mutual nearest neighbors (MNN) approach available from the batchelor R package was used to perform batch correction on top 2000 highly variable genes. Then, first 50 dimensions of the MNN low-dimensional corrected coordinates for all cells were used as input to produce the t-stochastic neighbour embedding (t-SNE) projection and uniform manifold approximation and projection (UMAP) using the “runTSNE” and “runUMAP” functions from the scater R package (v1.22.0) respectively. Putative cell clusters were identified using graph-based algorithms from the igraph R package (v1.3.0). Contaminating clusters, identified based on their expression of gene signatures associated with T cells, B cells, monocytes, and non-immune cells, were removed and remaining cells were re-clustered. For the analysis of publicly-available mouse single-cell RNA seq ^19^, we subsetted cells belonging to the control Id2^CreERT2^ population, and removed outliers and cells belonging the to the “unknown” cluster. Highly variable genes were identified and used to reduce the dimensions of the dataset using Principal Component Analysis (PCA). The top 10 Principal Components were used to plot reduce to two dimensions using t-SNE. SCENIC analysis ^31^ was performed as previously described ^19^.

#### Human Single Cell RNAseq

After sorting, single cell gene expression libraries were prepared using the Chromium Controller and Single Cell 5’ Reagent Kits v2 (10x Genomics) as indicated by the manufacturer’s instructions (CG000331 Rev E). Libraries were sequenced by SciLIfeLab (NGI; Stockholm, Sweden) using a NovaSeq 6000 sequencer and a SP-100 v1.5 flow cell (Illumina) with a read set up of 26 cycles for read 1, 10 cycles for the i7 index, 10 cycles for the i5 index, and 90 cycles for read 2. Count matrices were generated from FASTQ files using CellRanger v 7.0.1 (10X Genomics) and downstream analysis was performed using the R package Seurat (v 4.1) ^48^. Harmony was used for batch effects correction ^49^. For transcriptome analysis, Seurat was used for cell quality control, data normalisation, data scaling, dimension reduction (both linear and non-linear), clustering, differential expression analysis, and data visualization. Briefly, low quality cells were removed according to the number of detectable genes (number of genes < 200 or > 2500 were removed) and percentage of mitochondrial genes for each cell (≥5%). Data were normalised by a log-transform function with a scaling factor of 10,000. We used variable genes in principal component analysis (PCA) and used the top 10 batch effects corrected principal components (PCs) in non-linear dimension reduction and clustering. High-quality cells were then clustered by Louvain algorithm implemented in Seurat using the resolution of 0.5. Differentially expressed genes for each cell cluster were identified using a non-parametric Wilcoxon rank-sum test implemented in Seurat. Afterwards differentially expressed genes were used to identify clusters before removal of irrelevant clusters, including dead or dying cells and cytotoxic T cells. Remaining cells were then re-normalised and scaled and all variable genes were used in a second PCA. We then used the new top 8 batch effects corrected principal components in non-linear dimension reduction and clustering, with cells clustered by Louvain algorithm using the resolution of 0.5. Differentially expressed genes for each new cell cluster were again identified using a non-parametric Wilcoxon rank-sum and used to assign identities to the new clusters. These clusters were used for all downstream analysis and visualization. For ILC3 sub-clustering, the two clusters identified as “NKp44+ ILC3” and “NKp44- ILC3” were isolated from the rest of the dataset and re-clustered. We identified variable genes for this subset of cells and used the top 5 batch effects corrected principal components (PCs) in non-linear dimension reduction and clustering, with cells clustered by Louvain algorithm using a resolution of 0.5. Additional data visualizations were generated using the scCustomize package ^50^.

#### Microbiome sequencing and analysis

Transnetyx Microbiome kits containing barcoded sample collection tubes were provided by Transnetyx (Cordova, TN, USA). Mouse fecal samples were placed in individual tubes containing DNA stabilization buffer and shipped to Transnetyx (Cordova, TN, USA). DNA extraction was performed using the Qiagen DNeasy 96 PowerSoil Pro QIAcube HT extraction kit and protocol. Then, libraries were assembled using the KAPA HyperPlus library preparation protocol, and sequenced with the Illumina NovaSeq instrument and protocol, with a depth of 2 million (2x150 bp read pairs). Unique dual indexed (UDI) adapters were used to ensure that reads and/or organisms were not mis-assigned. FASTQ sequences were uploaded to the One Codex database of whole microbial reference genomes and aligned using k-mers, where k=31. Based on the relative frequency of unique k-mers in the sample, probable sequencing or reference genome artifacts were filtered out of the sample. Then, relative abundance of each species was estimated based on the depth and coverage of sequencing across every available reference genome. Principle coordinates analysis (PCoA) of microbial family β-diversity was performed using the Bray-Curtis method.

#### Quantification and statistical analysis

Bulk and single-cell RNAseq analysis was performed using R v4.2.0 and Bioconductor v3.13. All other analysis was performed using GraphPad Prism v.9.2.0. Details on statistical testing can be found in the corresponding figure legends. Use of parametric or non-parametric test was decided based on normal distribution of the datapoints, as determined using Shapiro-Wilk test. Significance was defined as *p<0.05, **p<0.01, ***p<0.001, ****p<0.0001.

## Supplementary Material

Supplementary Material

## Data Availability

Bulk RNA-seq data are available via NCBI’s Gene Expression Omnibus - accession number GSE228852. Murine single-cell RNA seq are available at ArrayExpress database - accession numbers E-MTAB-12864, and E-MTAB-9795 as previously published ^19^, and human single-cell RNA seq via NCBI’s Gene Expression Omnibus - accession number GSE229104. All other original data will be shared by the lead contact upon request. This paper does not report original code. Any additional information required to reanalyse the data reported in this paper is available from the lead contact upon request.
