# Supplementary material for: Bcl6 is a subset defining transcription factor of Lymphoid Tissue inducer-like ILC3
Source: Cell Rep. Author manuscript; Available in PMC 2024 Feb 15. (PMC7615641; doi:10.1016/j.celrep.2023.113425)
Supplement: Supplementary Material [file EMS193940-supplement-Supplementary_Material.docx]

**
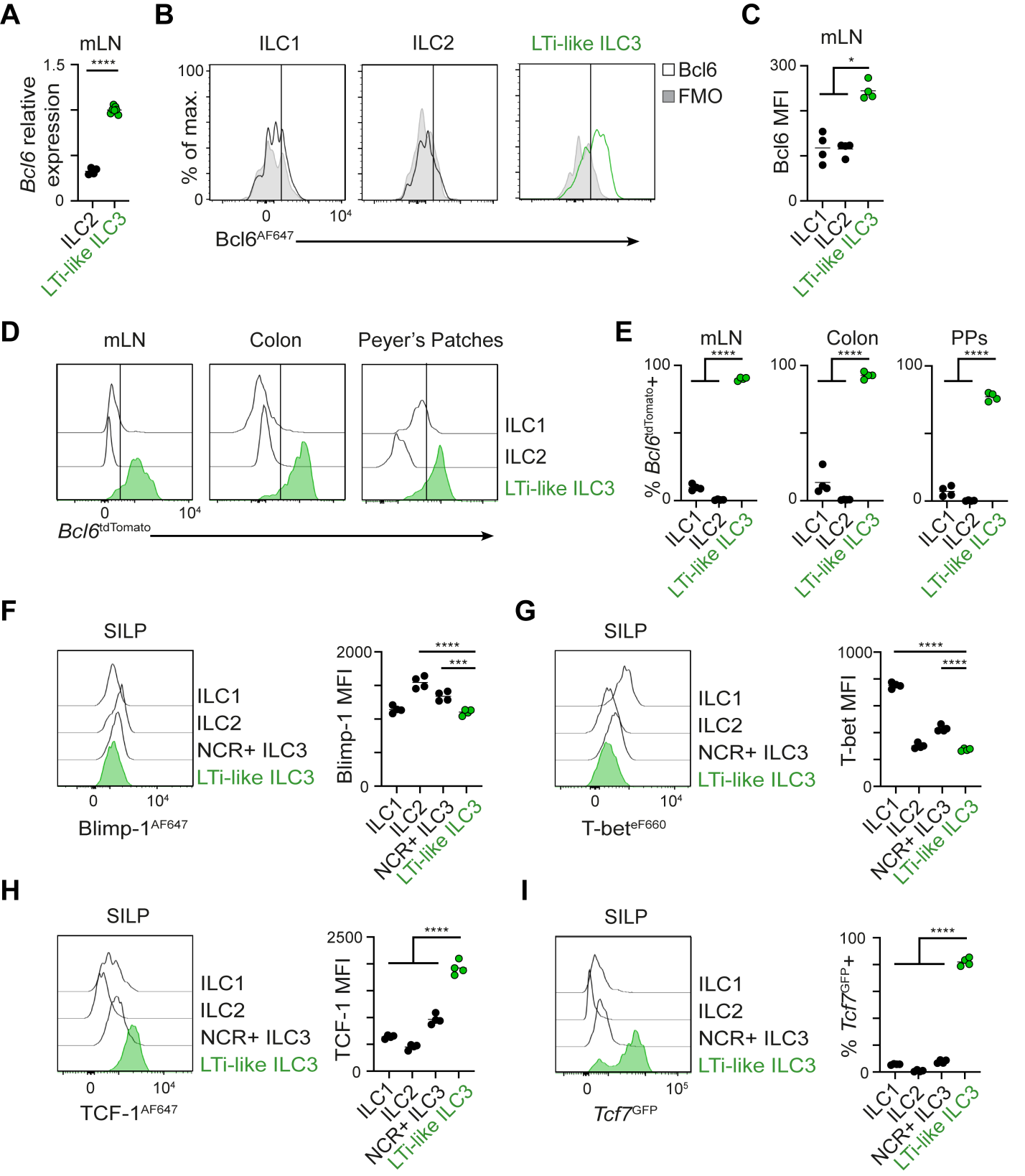
**

**Supplementary Figure 1. Bcl6 and the associated transcription factor TCF-1 are active in LTi-like ILC3,Related to Figure 1**. A) Relative expression of *Bcl6* mRNA in ILCs sorted from the mLN of RORγt^eGFP^ mice, determined by RT-PCR, n=4-7. B) Representative histograms and C) geometric mean quantification of Bcl6 protein across ILC subsets in mouse mLN, n=4. D) Representative histograms and E) quantification of the proportion of Bcl6^tdTomato^ expressing ILCs from the mLN, colon and PPs of Bcl6^tdTomato^ reporter mice, n=4. (F-I) Representative histograms and quantification of F) Blimp-1 G) T-bet, H) TCF-1 and I) *Tcf7*^GFP^ in SILP ILC subsets, n=4. Data pooled from 2 independent experiments (A), or representative of 2-4 independent experiments (B-I). Unpaired t-test (A), Kruskal-Wallis (C), Mann-Whitney test (K) or one-way ANOVA (E-I) with Dunn’s (C) or Dunnet’s (E-I) multiple comparisons test. Data represented as individual animals and mean.Significance was defined as *p<0.05, ***p<0.001, and ****p<0.0001.

**
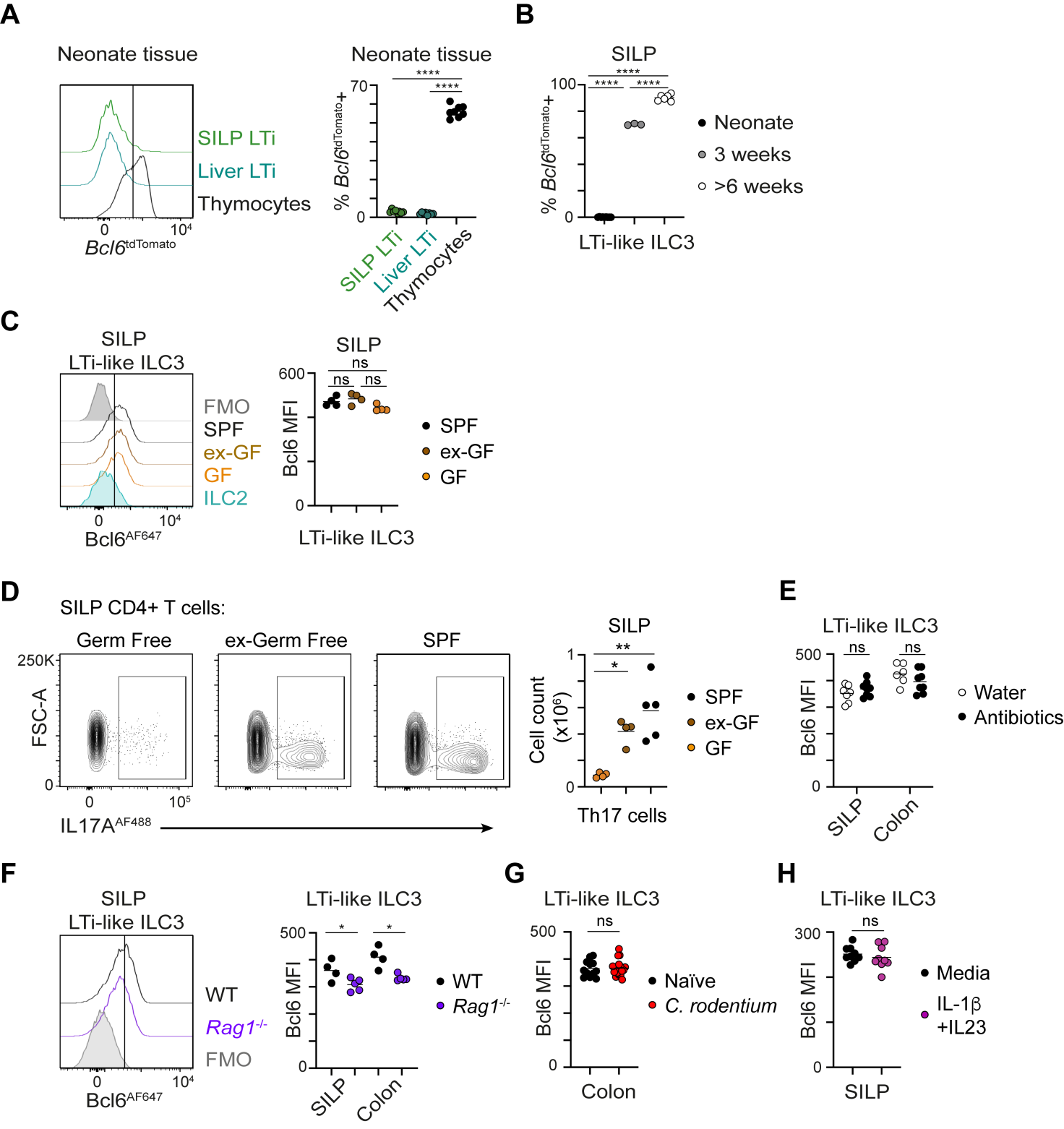
**

**Supplementary Figure 2. LTi-like ILC3 expression of Bcl6 is regulated postnatally but induced independently from microbial cues, Related to Figure 1**. A) Representative histograms (left) and quantification (right) of Bcl6^tdTomato^-expressing SILP LTi, liver LTi or thymus cells from neonate (1 day old) Bcl6^tdTomato^ reporter mice, n=8. B) Quantification of Bcl6^tdTomato^-expressing LTi-like ILC3 in the SILP of neonate (1 day old), 3-week-old or >6 week old Bcl6^tdTomato^ reporter mice, n=3-6. C) Representative histograms and quantification of Bcl6 expression by ILCs from SILP of Germ Free (GF), ex-GF, or specific pathogen-free (SPF) mice, n=4-5. D) Representative flow cytometry plots and quantification of the frequency of IL-17A-expressing CD4+ T cells in the SILP of mice as in (C). E) Quantification of Bcl6 expression on LTi-like ILC3 from the SILP or colon of mice treated with broad spectrum antibiotics or water, n=6-8. F) Representative histograms and quantification of Bcl6 expression by LTi-li3ke ILC from SILP or colon of *Rag1*^-/-^ or *Rag1*^+/+^ control (WT) mice, n=4-5. G) Quantification of Bcl6 expression on LTi-like ILC3 from the colon of mice infected with *Citrobacter rodentium*, n=12-18. H) Quantification of Bcl6 expression on SILP LTi-like ILC3 stimulated *ex vivo* with IL-1β and IL-23, n=9. Data representative of 2 independent experiments (A-D, F) or pooled from (E, H), or three (G) 2 independent experiments. Significance was calculated with a one-way ANOVA (A-B, D), a Kruskal-Wallis test (C), an unpaired t test (E, G), a Mann-Whitney test (F), or a paired t test (H). Data represented as individual animals and mean. Significance was defined as *p<0.05, **p<0.01 and ****p<0.0001.

**
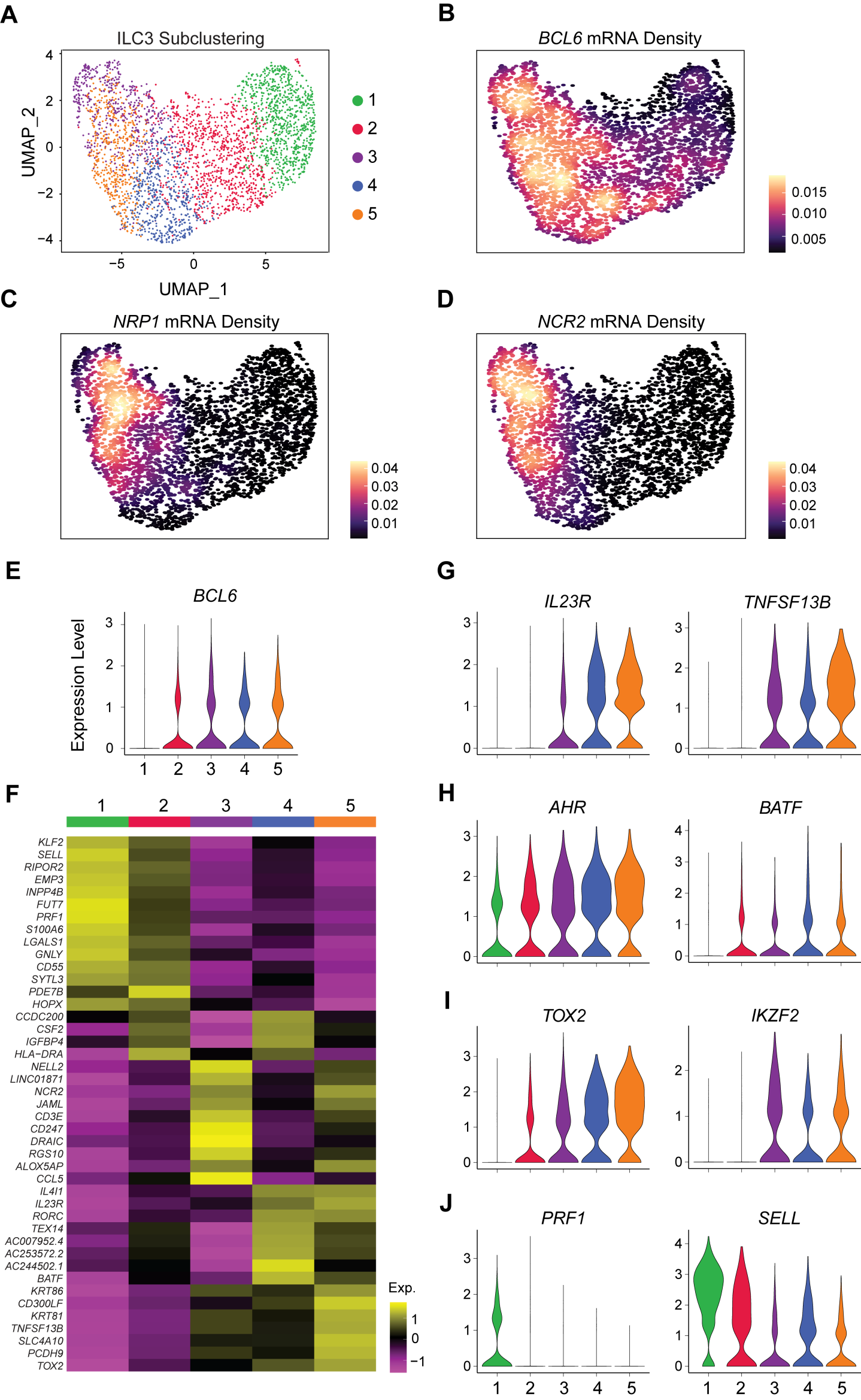
**

**Supplementary Figure 3. Transcriptional analysis of human ILC3 expressing *BCL6*, Related to Figure 2**. A) UMAP showing ILC3 sub-clusters from single-cell RNA seq data generated from human tonsil derived cells (from Figure 2). B) *BCL6*, C) *NRP1*, and D) *NCR2* mRNA expression density in ILC3 sub-clusters. E) Violin plot summarizing *BCL6* expression from ILC3 clusters 1-5. F) Heatmap showing expression of differentially expressed genes from ILC3 clusters. (G-J) Violin plots showing expression of G) ILC3-associated cellular communication genes - *IL23R* and *TNFSF13B*, H) transcription factors - *AHR* and *BATF*, I) *TOX2* and *IKZF2*, and J) phenotypic markers and effector molecules - *PRF1* and *SELL*.

**
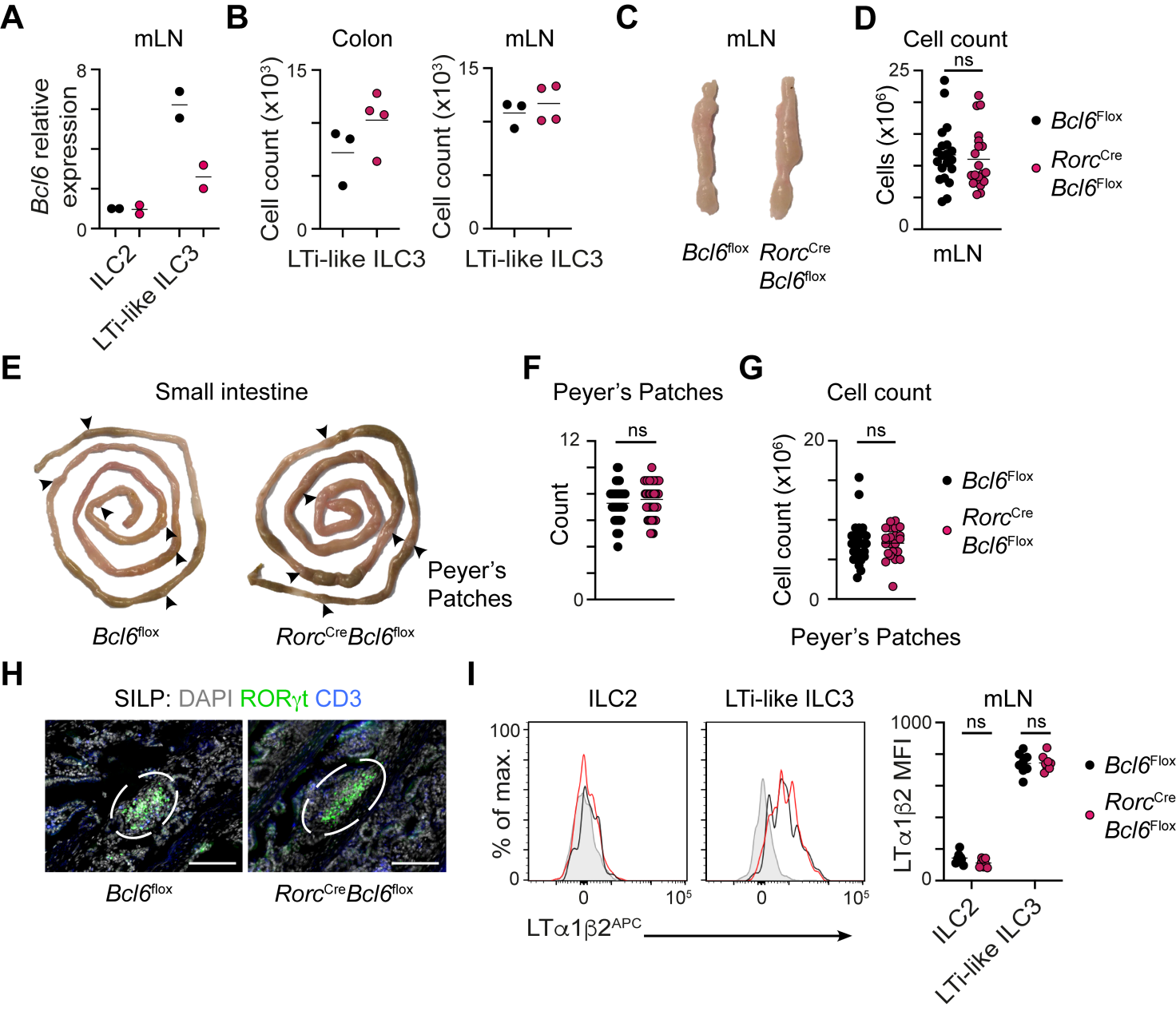
**

**Supplementary Figure 4**. **Bcl6 is dispensable for LTi-like ILC3 roles in lymphoid organogenesis, Related to Figure 3**. A) Relative *Bcl6* expression determined by RT-PCR on sorted cells from the mLN of *Rorc*^Cre^ x *Bcl6*^fl/fl^ and *Bcl6*^fl/fl^ control mice, n=2. B) mLN and colon LTi-like ILC3 absolute cell counts, n=3-4. C) Representative images of the mLN and D) total mLN cell counts from *Rorc*^Cre^ x *Bcl6*^fl/fl^ and *Bcl6*^fl/fl^ control mice, n=20. E) Images of the small intestine of *Rorc*^Cre^ x *Bcl6*^fl/fl^ and *Bcl6*^fl/fl^ control mice. Arrowheads indicate Peyer’s Patches (PP). F) Total count per mouse (n=43-44) and G) numbers (n=22-26) of total PP-associated cells from *Rorc*^Cre^ x *Bcl6*^fl/fl^ and *Bcl6*^fl/fl^ control mice. H) Immunofluorescence microscopy image of small intestine sections of *Rorc*^Cre^ x *Bcl6*^fl/fl^ and *Bcl6*^fl/fl^ control mice, stained for nuclei (grey), RORγt (green) and CD3 (blue). Dashed lines indicate cryptopatch architecture. Scale bar 100μm. I) Representative histograms and fluorescence intensity quantification of LTα1β2 on ILC from the mLN of *Rorc*^Cre^ x *Bcl6*^fl/fl^ and *Bcl6*^fl/fl^ control mice, n=8. Data pooled from (A, I) two, (D) five, (F) twelve (G) or seven independent experiments, or representative of a single experiment from (B) five or (H) two independent experiments. Significance calculated using an unpaired t test (B, I) or Mann-Whitney test (D-G). Data represented as individual animals and mean.

**
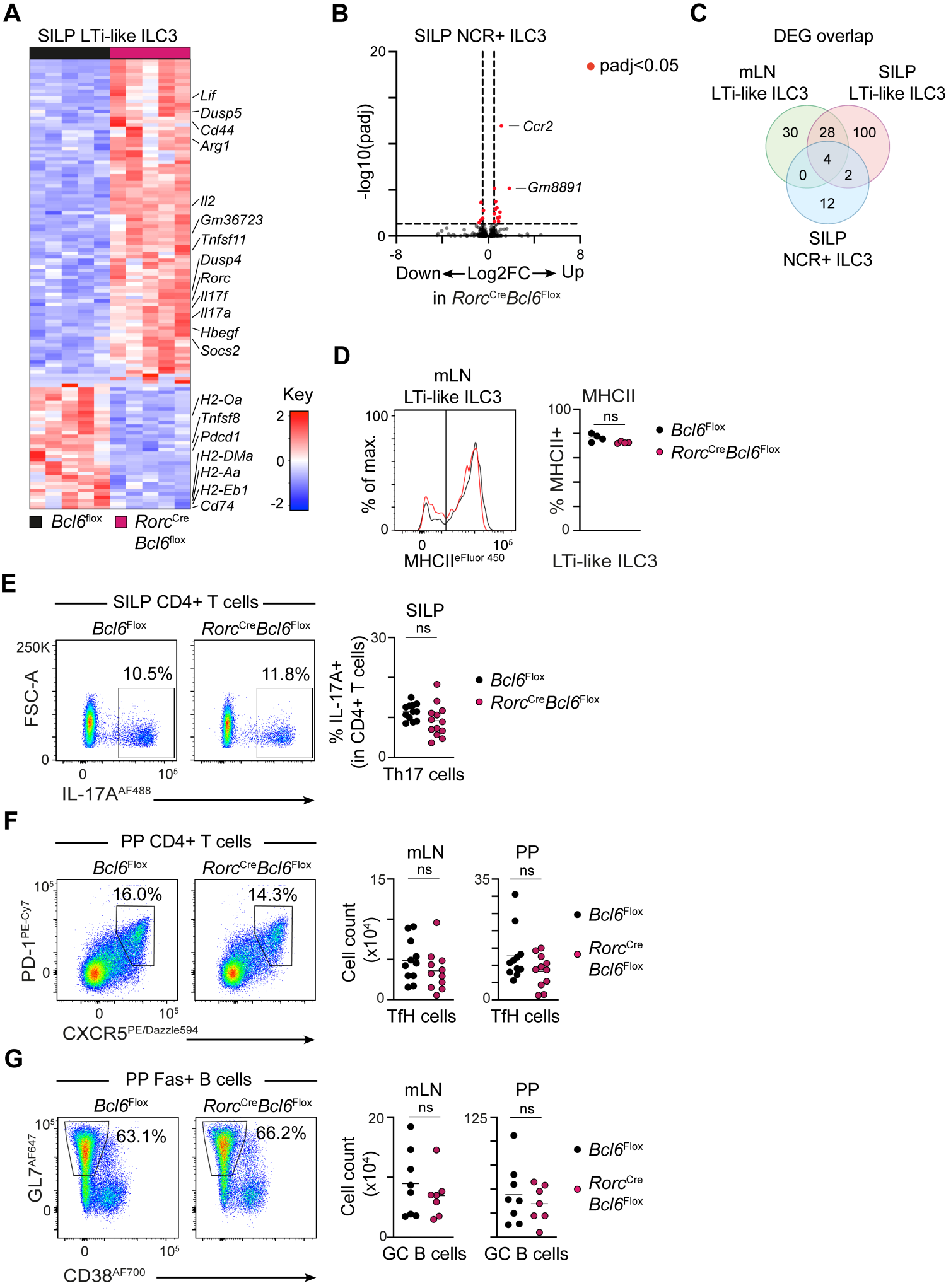
**

**Supplementary Figure 5. Bcl6-deletion perturbs the LTi-like ILC3 transcriptome, Related to Figure 3**.A)Heatmap showing differentially expressed genes between LTi-like ILC3 from the SILP of *Rorc*^Cre^ x *Bcl6*^fl/fl^ and *Bcl6*^fl/fl^ control mice. B) Volcano plot summarizing gene fold change (Log2FC) and adjusted P value, as -log10(padj), on NCR^+^ ILC3 of the SILP. C) Venn diagram showing differentially expressed gene numbers and overlap between LTi-like ILC3 from the mLN (green), or SILP (pink), or NCR^+^ ILC3 from the SILP (blue). D) Representative flow cytometry histogram and quantification of MHCII expression on LTi-like ILC3 from the mLN from *Rorc*^Cre^ x *Bcl6*^fl/fl^ and *Bcl6*^fl/fl^ control mice, n=4. (E-G) Representative flow cytometry histograms and quantification of E) SILP IL-17A+ CD4+ Th17 cells (n=12-13), F) PP PD-1+ CXCR5+ TfH cells (n=11) and G) PP GL7+ CD38- Germinal Center B cells (n=7-8), from *Rorc*^Cre^ x *Bcl6*^fl/fl^ and *Bcl6*^fl/fl^ control mice. Data representative of five independent experiments (D), or pooled from three (E-F) or two (G) independent experiments. Significance calculated using an unpaired t test (D, G) or Mann-Whitney test (F). Data represented as individual animals and mean.

**
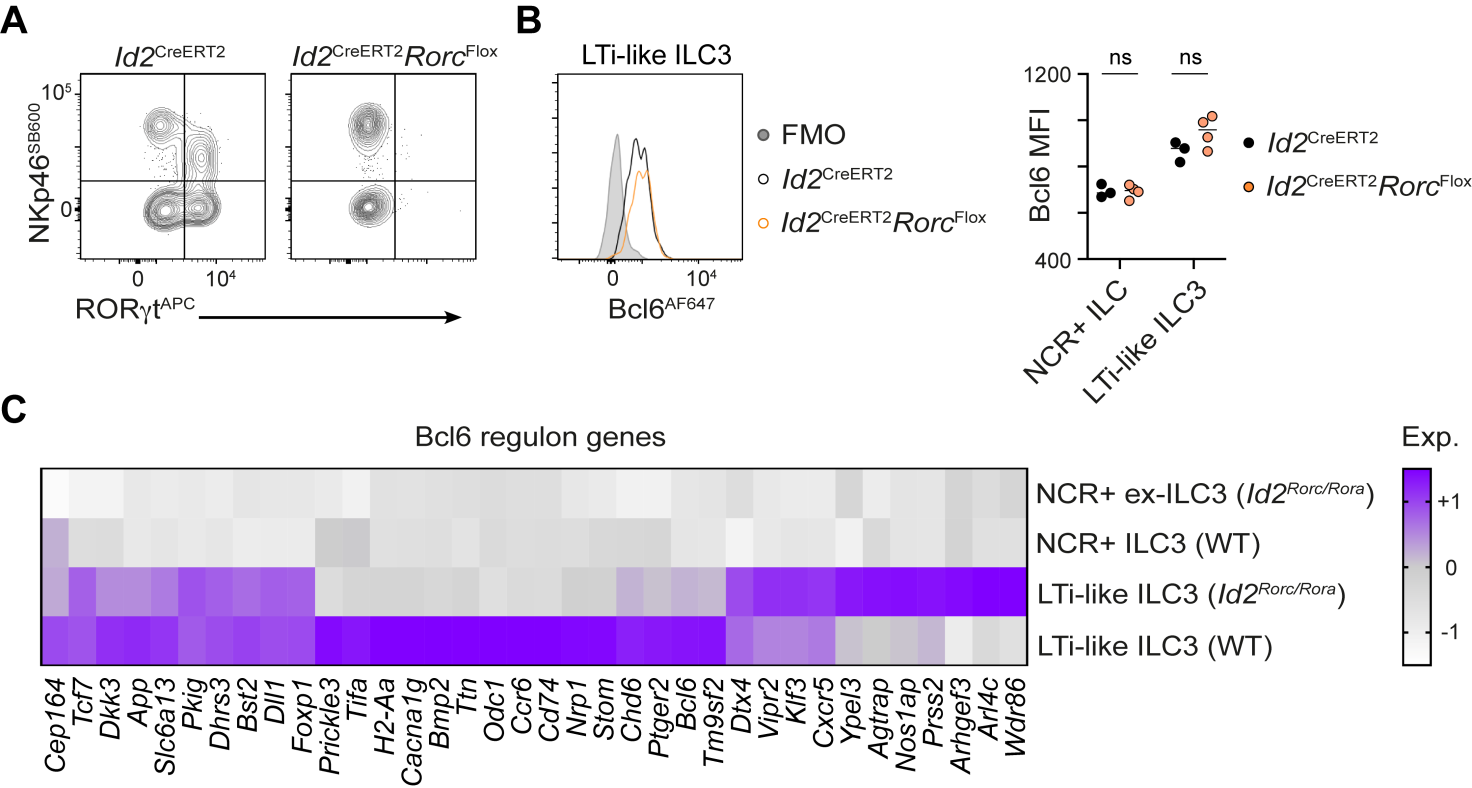
**

**Supplementary Figure 6. NCR+ ILC3, ex-ILC3 and ILC1 (“NCR+ ILC”) do not express Bcl6 or its associated regulon, Related to Figures3 and 4**. A)Representative flow cytometry plots showing expression of NKp46 and RORγt within RFP+ ILC from the SILP of *Id2*^CreERT2^ and *Id2*^CreERT2^ x *Rorc*^fl/fl^ mice. B) Representative flow cytometry histogram showing expression of Bcl6 in NCR+ ILC and LTi-like ILC3 from the SILP of mice as in (A), n=3-4. C) Heatmap showing the relative expression of *Bcl6* regulon-associated genes by NCR+ ILC and LTi-like ILC3 subsets from scRNA seq analysis of SILP ILC from *Id2^Rorc/Rora^* and control *Id2*^CreERT2^ (WT) mice. Data representative of three independent experiments (A-B).

**
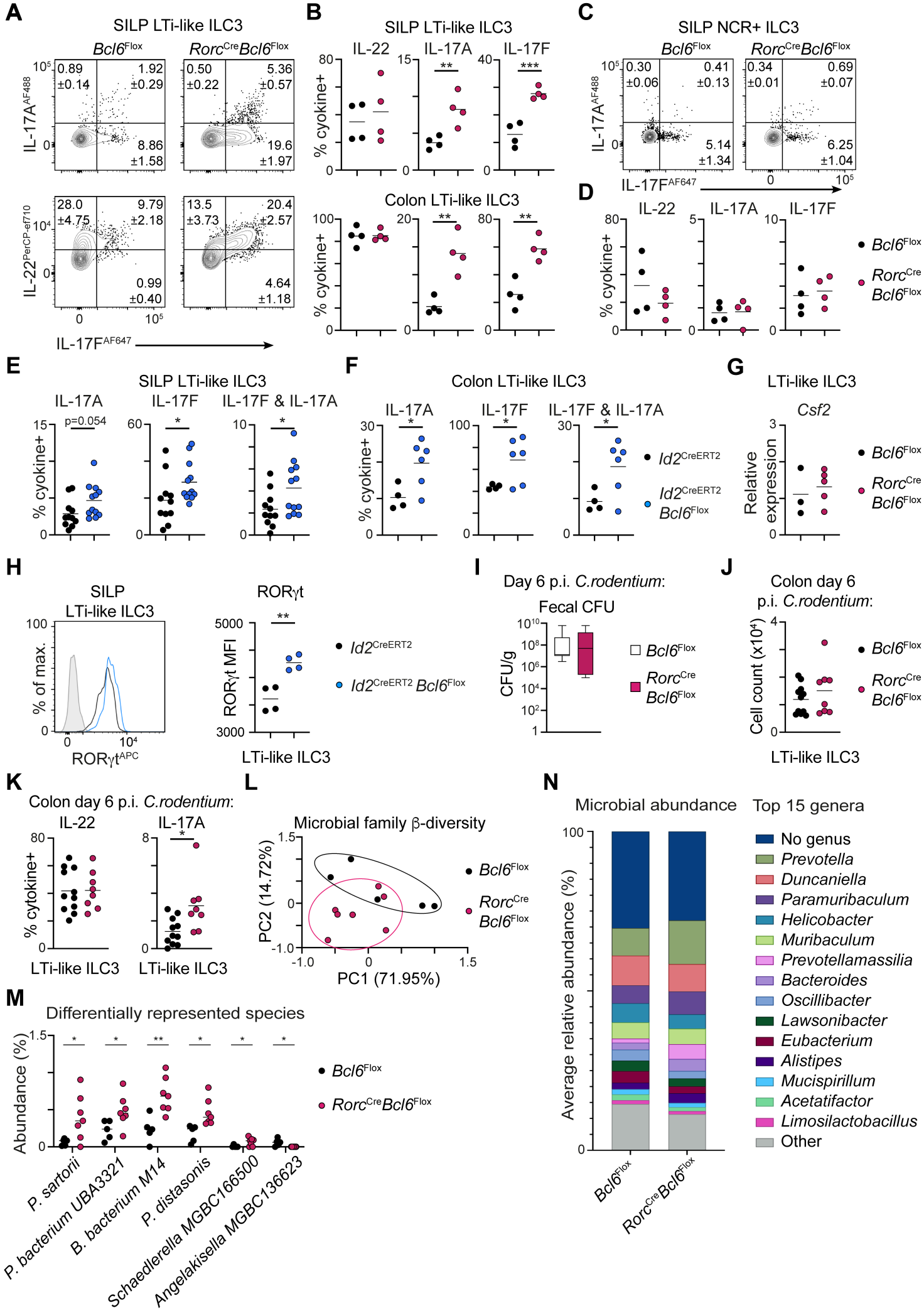
**

**Supplementary Figure 7. Effects of Bcl6 deletion on ILC3 cytokine production and anti-microbial responses, Related to Figure 5**. A) Representative flow cytometry plots and B) quantification of IL-17A, IL-17F and IL-22 production by ex-vivo stimulated LTi-like ILC3 from the SILP and colon, or C+D) SILP NCR^+^ ILC3 of *Rorc*^Cre^ x *Bcl6*^fl/fl^ and *Bcl6*^fl/fl^ control mice, n=4. Numbers on plots indicate average frequency (± standard error of the mean) of each quadrant. (E-F) Frequency IL-17A^+^, IL-17F^+^, and IL-17A^+^IL-17F^+^ double-producing LTi-like ILC3 from E) the SILP (n=11-12) and F) the colon (n=4-6) of *Id2*^CreERT2^ and *Id2*^CreERT2^ x *Bcl6*^fl/fl^ mice. G) Relative *Csf2* expression determined by RT-PCR on sorted cells from the SILP of *Rorc*^Cre^ x *Bcl6*^fl/fl^ and *Bcl6*^fl/fl^ control mice, n=3-5. H) Representative histogram (left) and geometric mean quantification (right) of RORγt in LTi-like ILC3 from the SILP of mice as in (E), n=4. (I) Fecal *C. rodentium* CFU counts, and J) absolute LTi-like ILC3 counts from the colon on day 6 post-infection in *Rorc*^Cre^ x *Bcl6*^fl/fl^ and *Bcl6*^fl/fl^ control mice, n=8-11. K) IL-22 and IL-17A production by LTi-like ILC3 from the colon of *C. rodentium* infected mice, n=8-11*.* L) Principal Coordinate Analysis of fecal microbial family β-diversity in naïve *Rorc*^Cre^ x *Bcl6*^fl/fl^ and *Bcl6*^fl/fl^ control mice, n=5-7. M) Graph showing abundance of differentially represented species (p value<0.05) and N) stacked bar chart showing relative abundance of the 15 most abundant genera within the fecal microbiota of in naïve *Rorc*^Cre^ x *Bcl6*^fl/fl^ and *Bcl6*^fl/fl^ control mice, n=5-7. Data representative of 2-4 independent experiments (A-D, H), or pooled from two (G, I-K) or three (E) independent experiments. Statistical significance calculated using an unpaired t-test (C, D, F-H, J, M), or Mann-Whitney test (E, I, K). Whiskers in I define minimum to maximum values. Data represented as individual animals and mean.Significance was defined as *p<0.05, **p<0.01 and
